# Genome- and transcriptome-wide association meta-analysis reveals new insights into genes affecting coronary and peripheral artery disease

**DOI:** 10.1371/journal.pone.0335513

**Published:** 2025-11-18

**Authors:** Michael Rode, Maciej Rosolowski, Katrin Horn, Sylvia Henger, Andrej Teren, Kerstin Wirkner, Joachim Thiery, Markus Loeffler, Janne Pott, Holger Kirsten, Markus Scholz

**Affiliations:** 1 Institute for Medical Informatics, Statistics and Epidemiology, University of Leipzig, Leipzig, Germany; 2 LIFE Research Center for Civilization Diseases, University of Leipzig, Leipzig, Germany; 3 Bielefeld Municipal Hospitals, Bielefeld, Germany; 4 Medical Campus Kiel, University Hospital Schleswig-Holstein, Kiel, Germany; 5 University of Cambridge, MRC Biostatistics Unit, Cambridge, United Kingdom; 6 Center for Scalable Data Analytics and Artificial Intelligence (ScaDS.AI) Dresden/Leipzig, Dresden, Germany; Calico: Calico Life Sciences LLC, UNITED STATES OF AMERICA

## Abstract

**Background:**

A low ankle-brachial Index (ABI) is an established condition for peripheral artery disease (PAD) and cardiovascular disease risk. The search for genetic determinants of the ankle-brachial index (ABI) is important to better understand molecular patho-cmechanisms of PAD and its commonalities with cardiovascular diseases (CVD), supporting development of new drug targets and tailored preventive or therapeutic measures.

**Methods:**

To search for genetic factors contributing to ankle-brachial index, we integrated genome-wide association meta-analysis and transcriptome-wide association meta-analysis (TWAMA) of two German cohorts, the population-based LIFE-Adult cohort and LIFE-Heart, a cohort of patients with suspected or confirmed coronary artery disease. Pathway analysis of identified genes was used to explore biological mechanisms potentially involved in ABI pathophysiology. Finally, we analysed co-associations of known CAD or carotid plaque associations with ABI to detect possible genetic commonalities.

**Results:**

By our GWAS meta-analysis, we identified four new gene loci associated with ABI that are also linked with coronary artery diseases (CAD) (6q26: *LPA* and 11q14.1: *DLG2*) or cholesterol levels (12q21.31: *TMTC2* and Xp21.1: *DMD*). Furthermore, we replicated a known ABI locus on cytoband 9p21.3 (*CDKN2B*) and four loci associated with PAD. In our TWAMA, we identified 145 blood transcripts associated with ABI at FDR 5% level. Gene set enrichment analysis of all TWAMA results revealed the inflammation-related pathways *interferon gamma response*, *neutrophil degranulation*, and *interferon alpha response* as the top three upregulated pathways in patients with lower ABI. Among overlapping genes between blood TWAMA and tissue-specific genetically regulated gene-expression association analysis, 24 genes showed consistent effect directions at nominal significance, with lower ABI-associated genes relating to stress response and vascular integrity, while higher ABI-associated genes linked to cellular homeostasis and metabolism.

**Conclusions:**

In our integrated genome- and transcriptome-wide meta-analysis, we identified novel and confirmed known candidate genes and pathways associated with ABI. Association signals partly overlap with those of other cardiovascular traits such as CAD and carotid plaque formation. The integration of gene-expression data, validated known and added new molecular insight how inflammatory signalling can contribute to atherosclerosis and vascular dysfunction. These findings pave the way for improved understanding of the molecular underpinnings of PAD and inform future strategies for targeted prevention and therapy.

## Introduction

The Ankle Brachial Index (ABI) is an important non-invasive assessment of peripheral arteries and is used to screen for peripheral artery disease and general cardiovascular risk [1]. The ABI is calculated by dividing the systolic blood pressure at the ankle by the systolic blood pressure in the arm. By this normalization of peripheral vs. more central blood pressure, ABI is considered a marker of peripheral artery disease (PAD) at the lower extremities. This non-invasive test requires only a blood pressure cuff and Doppler device, or, as in one of our studies, use oscillometric devices, making it accessible in clinical settings. Previous GWAS of ABI include the international Cohorts for Heart and Aging Research in Genomic Epidemiology (CHARGE) Consortium (n > 16,000) identifying rs10757269 at 9p21 [2], and recent work in the Million Veteran Program (n > 31,000) identifying 18 novel PAD loci [3]. Our study extends these findings using integrated genomic and transcriptomic approaches in well-phenotyped German cohorts: LIFE-Adult, a population- based study of 10,000 participants, and LIFE-Heart, comprising patients with cardiovascular disease. Normal ABI ranges from 1.00 to 1.40, ABI < 0.90 is closely related to the incidence of Peripheral arterial disease (PAD) while ABI > 1.40 indicates calcified or non-compressible vessels [1]. ABI has a high sensitivity in detecting PAD [4], while the specificity becomes lower in populations with certain conditions like Coronary Artery Disease (CAD) [5] or chronic kidney disease (CKD) [6]. Furthermore, ABI is also a predictor for the long-term cardiovascular health of a patient and can be used as first-line screening test for CAD because of its high specificity [7]. ABI values ≤0.90 are associated with an increased risk of major cardiovascular events, namely mortality, acute myocardial infarction, and ischemic stroke [8] and ABI values >1.40 are associated with an increased risk of all-cause mortality [9].

To better elucidate the molecular mechanisms affecting ABI, we integrate genome-wide genetic and transcriptomic association meta-analyses using measured or tissue-specific genetically inferred gene-expression profiles of two cohorts. Based on these findings, we perform pathway enrichment analysis to identify groups of genes associated with ABI and compare the findings of all three approaches on the gene and pathway level.

## Materials and methods

### Cohort description

We meta-analyse two studies collected in the LIFE Research Center for Civilization Diseases of the University of Leipzig, Germany. The LIFE-Adult study is a population-based cohort study of 10,000 participants from the city of Leipzig [10]. The majority of individuals in the study fall within the age range of 40–79, while a smaller subset of 400 participants are aged between 18 and 39. The study group consists of individuals of central European ancestry, and the primary aim of the study is to examine the prevalence, genetic predisposition, and influence of lifestyle-related factors (such as smoking, alcohol consumption, diet, and physical activity) on major chronic diseases, including subclinical markers of cardiovascular disease. The initial data collection for the study took place in between 2011 and 2014.

LIFE-Heart is an observational study of patients collected at the Heart Center of Leipzig, Germany, which is one of the worldwide leading Heart centers with a recruitment area covering several German federal states. The study design and a detailed description of patients can be found elsewhere [11]. A total of 6,994 patients were recruited with suspected or confirmed stable coronary artery disease (CAD) or myocardial infarction. Initial data collection was performed in between 2006 and 2014.

Baseline characteristics of both cohorts are provided at Table 1.

**Table 1:** Study characteristics of LIFE-Adult and LIFE-Heart.

Study characteristics		
***Parameter***	***LIFE-Adult (n = 6,508)***	***LIFE-Heart (n = 3,154)***
Men/ Women	3,167/ 3,341	2,018/ 1,136
Age (years)	57.5 ± 12.5	62.1 ± 10.7
Smoker/ Non-Smoker	1,360/ 5,148	654/ 2,500
Systolic blood pressure (mmHG)	128.7 ± 16.8	137.4 ± 18.8
Diastolic blood pressure (mmHG)	75.4 ± 9.8	83.3 ± 11.1
Peripheral Arterial Disease (ABI ≤ 0.9)	362 (5.6%)	348 (11.0%)
Diabetes (HbA1c > 6.5%)	1,099 (16.9%)	1,651 (52.3%)
ABI	1.08 ± 0.12	1.07 ± 0.17
Total cholesterol (mmol/l)	5.61 ± 1.07	5.45 ± 1.20
HDL-cholesterol (mmol/l)	1.62 ± 0.47	1.34 ± 0.41
LDL-cholesterol (mmol/l)	3.51 ± 0.96	3.34 ± 1.04

Continuous parameters are presented as mean ± standard deviation. Analysis is restricted to participants/ patients for which genotypes, ABI and smoking status are available. Former smokers and never smokers were summarized to non-smokers. Participants with ABI > 1.4 or on which percutaneous transluminal angioplasty had been performed were excluded.

Both studies meet the ethical standards of the Declaration of Helsinki and were approved by the Ethics Committee of the Medical Faculty of the University Leipzig, Germany (LIFE-Adult: Reg. No 263-2009-14122009; LIFE-Heart: Reg. No. 276–2005). Written informed consent including agreement with molecular-genetic analyses was obtained from all participants. Data were accessed in December 2019. Authors had no access to information that could identify individual participants during or after data collection.

### ABI assessment

ABI was determined taking into account the recommendation of the American Heart Association [12]. For LIFE-Adult, blood pressure was measured using oscillometry-, and photoplethysmography-based methods (Vicorder, Skidmore medical, UK) [13]. For both sides, we averaged the three available systolic blood pressure (SBP) measurements for each ankle and each upper-arm separately. We then calculated the ABI for the right side (left side) by dividing the average value for the right ankle (left ankle) by the higher value of the average values for the right and the left upper-arm. The lower of the two quotients was used as ABI assessment for the respective participant.

For LIFE-Heart blood pressure was measured by Doppler sonography sphygmomanometer cuffs and a hand-held Doppler probe (Huntleigh Mini-Dopplex, Germany) [11]. We averaged the two available SBP measurements for each ankle. For the upper-arms we only had two SBP measurements for the right side which we also averaged. ABI for the right side (left side) was calculated by dividing the average value for the right ankle (left ankle) by the average SBP of the upper-arm. The lower of the two results was considered as ABI for the respective participant.

For both cohorts, participants with ABI > 1.4 were removed as ABI values above 1.4 indicate calcified vessels most common in patients with diabetes [14]. We also removed participants with percutaneous transluminal angioplasty or without genetic data.

### Genotyping

Both LIFE studies were genotyped using the Affymetrix Axiom SNP-array technology [15] (LIFE-Adult: CEU1 array, LIFE-Heart: CEU1 or CADLIFE array, a customized CEU1 array containing additional SNPs from CAD loci). For each study genotype calling was performed with Affymetrix Power Tools (v1.20.6 for LIFE-Adult CEU1; v1.17.0 for LIFE-Heart CADLIFE; v1.16.1 for LIFE-Heart CEU1), following best practice steps for quality control. These steps comprised filtering samples for signal contrast and sample-wise call rate, and included SNP filters in regards to platform specific cluster criteria. The LIFE-Heart datasets from the two distinct array platforms were combined after calling [16].

Samples with XY irregularities, including sex-mismatches or cryptic relatedness, and genetic outliers (>6 standard deviations of genetic principal components) were excluded.

Cryptic relatedness was assessed using pairwise relatedness estimators [17]. Pairs of individuals exceeding a relatedness threshold > 0.2, corresponding to second-degree relatives, were identified. In total, 243 such pairs were detected in LIFE-Adult and 208 in LIFE-Heart. To maximize statistical power, related individuals were not excluded per se; removal occurred only for additional quality control issues (e.g., sex mismatch or duplicated measurements), since it was demonstrated that inclusion does not relevantly affect association results [18] for situation with inflation factors below 1.05, which we confirmed by a sensitivity analysis (see section Statistical analysis). In total, 11 individuals in LIFE-Adult and 118 in LIFE-Heart were excluded due to relatedness in combination with other QC issues, 37 and 6 due to outlying principal components (mean ± 6 SD on first 10 PCs), 6 and 15 due to sex mismatches and 9 and 12 for analysis of chromosome X due to XY anomalies, respectively. To ensure unbiased ancestry inference, principal component analysis (PCA) was initially performed on a subset of unrelated individuals (all pairwise relatedness estimators < 0.05). Subsequently, related individuals were projected into the PCA space using the “swap-one-in PCA” approach, whereby each related individual was added one at a time to the unrelated set to estimate their principal components without affecting the overall population structure. The PCA was done with PLINK 1.9.

Further, variants with a call rate less than 0.97 and Hardy-Weinberg Equilibrium P < 1x10^-6^, were removed before imputation. Imputation was performed using the 1000 Genomes Project phase 3, Version 5, European reference panel [19] and IMPUTE2 [20] as software. In summary, 7,669 and 5,700 samples were successfully genotyped in LIFE-Adult and LIFE-Heart, respectively (7,660 and 5,688 samples for chromosome X). After filtering subjects with non-valid ABI measurement as described above, 6,508 and 3,154 individuals were included in our genetic association study, respectively.

### Gene expression analysis

For the LIFE-Adult study, whole blood was collected in Tempus Blood RNA Tubes (Life Technologies) and relocated to −80°C before RNA isolation. For the LIFE-Heart study, RNA was extracted from peripheral blood mononuclear cells (PBMCs, see Burkhardt et al. [21] and Holdt et al. [22] for a complete description of the sampling and measurement process).

Human HT-12.0 Version 4 Expression BeadChips (Illumina, San Diego, CA, USA) were used to measure gene expression in blood samples from the LIFE-Adult (whole blood) and the LIFE-Heart cohorts (PBMC). Both data sets were preprocessed separately using a similar procedure. Raw gene expression data was extracted using Illumina GenomeStudio without background correction. Expression values were log2-transformed and quantile-normalized [23]. Batch effects of expression BeadChips were corrected using the ComBat method [24], which uses empirical Bayes frameworks to adjust for known batch variables while preserving biological variation. Success of adjustment was checked using ANOVA of the Sentrix barcode, as well as the processing batch (in a processing batch, several expression chips were jointly processed, in consequence, within a processing-batch, several Sentrix barcodes are completely nested). In LIFE-Heart, a total of 615 (1.3%) and in LIFE Adult, a total of 1,319 (2.7%) gene-expression probes still over-inflated following Bonferroni-correction were excluded from following preprocessing steps.

A total of 47,231 gene-expression probes are available on the array. Probes detected by Illumina GenomeStudio as expressed in less than 5% of the samples, showing associations with batch effects even after batch correction (with Bonferroni-adjusted p-value < 0.05) or without mapping to a gene according to IPA (ingenuity pathway analysis [25], were excluded. As a result, 36,374 (LIFE-Adult) and 36,368 (LIFE-Heart) gene-expression probes corresponding to 22,644 (22,641) genes remained in the data. From these probes, 36,366 corresponding to 22,640 genes were available in both data sets based on their probe IDs and were used in the meta-analysis.

With respect to gene-expression sample quality, we removed samples based on the following two conditions. First, the number of detected gene-expression probes of a sample had to be within ±3 interquartile ranges (IQR) from its median over all samples. Secondly, the Mahalanobis distance of several quality characteristics of a sample (signal of AmbionTM ERCC Spike-In control probes, signal of biotin-control-probes, signal of low-concentration control probes, signal of medium-concentration control probes, signal of mismatch control probes, signal of negative control probes and signal of perfect-match control probes) must not exceed the median + 3 IQR [26].

Overall, of the assayed 3,526 LIFE-Adult samples, 107 samples were excluded for quality reasons. Exclusion of the remaining duplicate samples (2 technical, 34 biological), samples that were not genotyped (189), samples with missing values of ABI, sex, age, smoking status, proportion of lymphocytes or proportion of monocytes (426), and patients with non-valid ABI measurement as described above (15) resulted in 2,753 samples available for further analysis.

Of the assayed 4,509 LIFE-Heart samples, 122 samples were excluded for quality reasons. Exclusion of duplicate samples (13), not genotyped samples (37), samples with missing values of ABI, sex, age, smoking status, proportion of lymphocytes and proportion of monocytes (1,766, of which 1,759 had missing ABI measurements), and patients with non-valid ABI measurement as described above (53), resulted in 2,518 samples ready for analysis.

To address differences between whole blood (LIFE-Adult) and PBMC (LIFE-Heart) expression profiles, we 1) Applied cell-type deconvolution using CIBERSORT to estimate and adjust for cell composition differences to account for non-available cell type composition; 2) Used separate normalization pipelines optimized for each sample type before meta-analysis; 3) Included estimated neutrophil percentage as a covariate since neutrophils are present in whole blood but not PBMCs; 4) Performed sensitivity analyses restricted to genes expressed in both sample types; and 5) Performed meta-analysis instead of pooling. Despite the differences in sample types, we observed high concordance in effect directions (85% for nominally significant genes), suggesting robust cross-sample-type associations.

### Statistical analysis

#### Genome-wide association meta-analysis.

We first performed genome-wide association analyses for ABI by linear regression models assuming an additive mode of inheritance with adjustments for sex, age and smoking status separately in each of the two LIFE studies using PLINK 2.0. We refrained from correction for population stratification due to low heterogeneity of study participants, and irrelevant association of population genetics principal components with the endpoint (S1 Table in S1 File, S1 Fig, S2 Fig). For LIFE-Adult (LIFE-Heart) only three (one) principal components were significantly correlated with a maximum explained variance of r^2^ = 1.6x10^-3^ for PC7 for LIFE-Adult (r^2^ = 2.7x10^-3^ for PC4 for LIFE-Heart).

All SNPs were harmonized between LIFE-Adult and LIFE-Heart to ensure the same effect allele. In addition, we checked for mismatching alleles or chromosomal position (hg19) with respect to 1,000 Genomes phase 3 European reference [19] and excluded SNPs with a high deviation of observed minor allele frequencies (absolute difference >20%). Only SNPs in the intersection of both studies were meta-analysed.

For genome-wide association meta-analysis, single study results per phenotype were combined using a fixed-effect model. We used I^2^ statistics to evaluate heterogeneity and filtered findings with I^2^ > 90%. Only SNPs with samples-size weighted average minor allele frequency (MAF) ≥ 1%, and a samples-size weighted average minimum imputation info-score of at least 0.8, analyzed in both studies, were used.

The threshold for genome-wide significance was set to p < 5x10^-8^. Associations with p < 1x10^-6^ were considered as suggestive. In order to determine independent hits, variants, which are in LD r^2^ ≥ 0.1 with a SNP with stronger p-value, were considered tagged by that SNP (priority pruning). Pruned suggestive hits were assigned to loci by defining a region of ±500 kb around each SNP. In our hands, this resulted in non-overlapping regions.

As a sensitivity analysis, we repeated the GWAS meta-analysis using two alternative approaches: (i) excluding one individual from all pairs of related samples, and (ii) including all individuals while adjusting for genetic population structure using the first ten principal components. Results from both approaches were highly consistent with the main analysis, indicating that cryptic relatedness and population structure were adequately controlled. Detailed comparisons are provided in S3 Fig. A comprehensive annotation was applied to all SNPs that reached at least suggestive significant association levels using the following bioinformatics resources: Physically nearby genes were looked up from Ensemble [27] within ±250 kb around a variant. For associated variants, we assigned other GWAS hits in LD (r^2^ > 0.3) retrieved from the GWAS catalogue [28] and previously reported as well as in-house determined expression quantitative trait loci (eQTL) [24–32]. Combined Annotation-Dependent Depletion (CADD) score was used to assess variant deleteriousness [29].

Furthermore, to look-up supporting evidence for our identified top-GWAS hits, we queried available GWAS and PheWAS resources, including the GWAS catalogue [28], dbGaP (https://www.ncbi.nlm.nih.gov/gdv/browser/dbgap-gwas/), Finngen freeze 5 (https://r5.finngen.fi/), UK Biobank PheWAS (https://pheweb.org/UKB-Neale/)/ GWAS [30], and CARDIoGRAMplusC4D [30].

Genome-wide summary statistics for GWAS meta-analysis are available at zenodo [31].

#### Replication and look-up of known SNPs associated with atherosclerotic diseases.

We looked up previously reported genetic loci for ABI, CAD, PAD and carotid plaque in the GWAS catalogue to replicate other reported ABI/PAD hits and to determine potential overlaps of genetic driving factors of ABI and other cardiovascular diseases as described in detail in Supplementary methods in S2 File. For reported ABI and PAD hits, we also compared concordance of reported effect sizes with our data. Summary statistics of other studies were retrieved from the GWAS catalogue [32]. From these studies we selected SNPs with associations that showed at least suggestive significance (p < 1x10^-6^). For each reported SNP, we identified all SNPs in our GWAS meta-analysis ±1Mb of the reported SNP with LD r^2^ ≥ 0.8. In the following, we proceeded phenotype-wise for GWAS-catalogue studies reporting on phenotypes ABI, CAD, PAD and carotid plaque: When multiple GWAS meta-analysis -SNPs were linked to the same GWAS-catalogue SNP, we selected the GWAS meta-analysis -SNP with the highest linkage disequilibrium (prioritizing direct SNP match over higher LD based on R^2^ over shorter physical). When multiple association studies were reported for the same GWAS-catalogue SNP, we selected the GWAS-catalogue study with the highest evidence (prioritizing lower p-value over higher sample size). To avoid biased replication rates due to the LD structure in our data, we considered only a single assignment of each GWAS-catalogue SNP to a certain GWAS meta-analysis SNP for all GWAS meta-analysis SNPs tagged by the same variant in our priority pruning approach described above. Thereby, we prioritized those GWAS-catalogue SNP – GWAS meta-analysis SNP pairs where the GWAS-catalogue studies had lower reported p-values over those linked with higher LD (based on R^2^) over those with shorter physical distance.

For the resulting SNP pairs, the effect allele and direction of effect were harmonized and compared between the literature and our meta-analysis results. We investigated replication at two levels: (1) a liberal criterion requiring nominal significance (one-sided p < 0.05) with the expected effect direction (i.e., reverse effect directions for ABI compared with CAD, PAD or carotid plaque), and (2) a more stringent criterion requiring significance after Benjamini-Hochberg false discovery rate (FDR) correction at 5% level, maintaining the same directional requirements. Since we primarily aimed at validating reported ABI or PAD associations, we estimated the respective statistical power of our study in this regard. We assumed that the true effect was as reported in the GWAS catalogue. Standard errors and effective sample sizes were also retrieved from this resource. We also assumed that the observed effect (regression coefficient of the ABI in our data) was normally distributed and that the minor allele frequencies of each SNP in the original study and in our study were equal. For power calculation regarding the binary trait PAD, we divided the reported effects by π/√3 = 1.81 as recommended [33] and multiplied the reported standard errors by$\sqrt{\frac{n_{orig}\cdot \phi (\text{1}-\phi )}{n_{rep}}}$, where φ denotes the proportion of PAD cases in the original study, and n_orig_ and n_rep_ denote the sample size in the original study and in our replication study, respectively [34].

#### Genetically-regulated gene-expression association analysis.

We used the S-PrediXcan method [35] to associate genetically predicted gene-expression of four tissues with ABI: aorta, coronary and tibial artery as well as whole blood. S-PrediXcan is a computational method than can use summary statistics from GWAS meta-analysis to predict gene expression levels of tissues based on genetic variants and to identify potential genetic associations of these inferred gene-expressions with other phenotypes. The method uses reference transcriptome data, which provides information on the expression levels of genes for different tissues, to construct prediction models of gene expressions based on genetic variants [36]. While we primarily analyse vascular tissue, blood was also analysed due to the superior expression quantitative trait loci (eQTL) models available for this tissue in GTEx [37]. Expression prediction models were downloaded from a respective GitHub repository [38] (see also PredictDB [39]). For data preparation, we lifted our data from hg19 to hg38 using the GWAS Summary Statistics harmonization tool [40]. Summary statistics of missing SNPs were imputed for the purpose of gene-expression prediction. We then tested the effects of gene expression variation on ABI in the four above mentioned tissues.

Genome-wide summary statistics for S-PrediXcan analysis are available at zenodo [31].

#### Transcriptome-wide association meta-analysis (TWAMA).

Gene-expression association analyses using directly measured blood transcriptomes were performed separately in the LIFE-Adult and LIFE-Heart cohort using limma [41]. Using the same linear model for both cohorts, each probe expression was treated as a response variable and ABI, sex, age, smoking status, proportion of lymphocytes, and proportion of monocytes as additive explanatory variables. In case lymphocyte and/or monocytes data were not available, these percentages were inferred using CIBERSORT [42] with percentage monocytes estimated as the difference between 100% and the sum of estimated neutrophils and lymphocytes. Results of single cohorts were again summarized by fixed-effect meta-analysis of the regression coefficients of ABI. Probes with I^2^ ≥ 50% were discarded and the p-values of the remaining probes were adjusted to control the false discovery rate (FDR) at 0.05 using the method of Benjamini and Hochberg [43]. Analyses were conducted in R version 4.1.1 [44].

Genome-wide summary statistics for TWAMA are available at zenodo [31].

### Pathway analysis

We performed separate pathway analyses using the results of three gene expression analyses: 1) S-PrediXcan results of the artery tissue (aorta, coronary and tibial), 2) S-PrediXcan of the whole blood tissue, and, 3) TWAMA-results from differential expression of the directly measured blood gene expression data. In case of multiple transcripts per gene, transcripts were summarized to unique gene symbols by selecting the transcript with the smallest meta-analysis heterogeneity (Cochran’s Q) for each gene to enrich for associations homogeneous between cohorts. Fast pre-ranked Gene Set Enrichment Analysis (FGSEA) [45,46] of lists of ranked genes was performed using the R packages fgsea and clusterProfiler [47]. In two separate analyses, enrichment of Disease Ontology (DO) [48,49], and, according to previously published recommendations [50], of gene sets representing pathways culled from several sources were computed. Those included GO (biological process, excluding annotations that have evidence code IEA, i.e., inferred from electronic annotation, excluding ND, i.e., no biological data available, and excluding RCA, i.e., inferred from reviewed computational analysis), Reactome, Panther, NetPath, NCI, IOB, WikiPathways, MSigDB C2, MSigDB Hallmark and HumanCyc, available at http://download.baderlab.org/EM_Genesets/April_01_2022/Human/symbol/Human_GO_AllPathways_no_GO_iea_April_01_2022_symbol.gmt. For S-PrediXcan of the artery tissue, genes were ranked by their z-scores that had the highest absolute value across the three artery tissues (aorta, coronary, tibial). For S-PrediXcan of the whole blood tissue, genes were ranked by their z-scores. For directly measured gene expression, genes were ranked according to their z-statistics of the fixed-effect meta-analysis of the ABI regression coefficients (see Section “Transcription-wide association meta-analysis”). Gene sets of size between 15 and 500 were included in the enrichment analyses (1005 gene sets from the DO and 546 gene sets representing pathways). Gene sets with Benjamini-Hochberg FDR-adjusted p-value < 0.05 were considered significant.

## Results

### Genome-wide association meta-analysis

**Fig 1:**
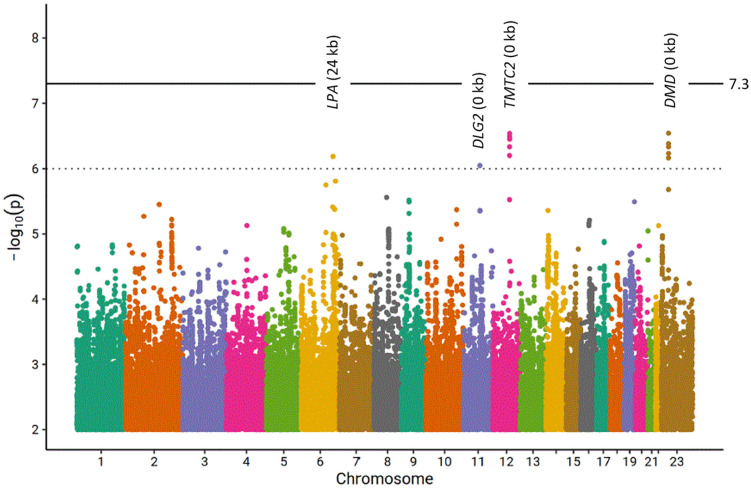
Manhattan plot of our genome-wide association analysis of ABI. Distribution of log10 transformed p-values. The bold line marks genome-wide significance (p < 5x10^-8^). Four loci show signals of suggestive significance (p < 1x10^-6^) but none of the signals achieved genome-wide significance. For each of these four loci, the nearest gene and the distance from the best associating SNP to this gene is reported.

After quality filtering, 8,828,968 SNPs remained for meta-analysis. No inflation of test statistic was observed (lambda = 0.9989, see S2 Fig for a quantile-quantile plot). No hits were found at genome-wide significance level, but four loci showed suggestively significant associating SNPs (see Table 2 for an overview and S2 table in S1 File for more detailed association statistics, Fig 1 for a Manhattan Plot for ABI and S5 Fig for regional association plots).

**Table 2:** Loci with suggestive evidence in our Meta-GWAS of ABI.

Cytoband	Top-SNP	ID_Meta (Build GRCh37)	Candidate gene (distance)	EA/ other allele	EAF	Info score	I²	Beta (SE)	p-value	I²	p-value random	Beta random (SE)
6q26	rs186696265	chr6:161111700:T:C	LPA (24 kb)	T/ C	0.02	0.94	0.61	−0.036 (0.007)	6.51x10^-7^	0.610	2.66x10^-3^	−0.041 (0.013)
11q14.1	rs200811106	chr11:84455545:TGG:T	DLG2 (0 kb)	TGG/ T	0.01	0.91	0	−0.042 (0.008)	8.98x10^-7^	0	7.95x10^-7^	−0.042 (0.008)
12q21.31	rs80115038	chr12:83244133:T:C	TMTC2 (0 kb)	T/ C	0.04	0.98	0	−0.023 (0.005)	2.90x10^-7^	0	2.51x10^-7^	−0.023 (0.005)
Xp21.1	rs5972515	chr23:32303187:A:G	DMD (0 kb)	A/ G	0.05	0.98	0.57	−0.018 (0.003)	2.87x10^-7^	0.576	1.25x10^-3^	−0.020 (0.006)

We present the four loci with suggestive evidence, its respective top-SNPs and association statistics, candidate genes EA: effect allele, EAF: weighted average of effect allele frequency across studies, info score: weighted imputation info score across studies. I^2^: Heterogeneity estimate of meta-GWAS.

We present the four loci with suggestive significant associations in more detail in the following. The lead SNP on cytoband 6q26 is rs186696265 (β = −0.036, p = 6.52x10^-7^) near *PLG* (12 kb) and *LPA* (24 kb). *LPA* is well known for controlling Lp(a) plasma concentrations also associating with a number of cardiovascular disease phenotypes [30,51] including PAD [52]. The SNP was identified in GWAS studies of multiple endpoints including CAD [30], cholesterol lowering medication (UK Biobank PheWAS), and cholesterol and triglycerides levels (S3 table in S1 File). The SNP was also in LD with several cis-eQTLs including the functionally plausible genes *ACAT2* involved in cholesterol metabolism [53], *SLC22A1* known for its role in elevated cholesterol and LDL-C levels [54] and *SOD2* linked to CAD [55]. Thus, this locus is functionally plausible but different candidate genes can be conceived.

On cytoband 11q14.1 the lead SNP is rs200811106 (β = −0.042, p = 8.98x10^-7^). This variant has a CADD score of 11.9 and is therefore likely to be deleterious. The SNP is in the coding sequence of *DLG2*, which has been associated with CAD before [56]. The SNP was also in linkage disequilibrium (LD) with variant rs142615018 in a GWAS associated with BMI (R^2^ = 0.74, S3 Table in S1 File).

On cytoband 12q21.31 the top-associated variant rs80115038 (β = −0.023, p = 2.90x10^-7^) is in the coding sequence of *TMTC2*. *TMTC2* has been associated with HDL levels before [57]. Although this variant is not in linkage disequilibrium with reported eQTL or CAD-associated large-scale GWAS [30], a large-scale PheWAS links it to the use of colestyramine, a cholesterol-lowering agent, supporting a role in lipid metabolism relevant to cardiovascular health [58].

The fourth locus is located at Xp21.1 with rs5972515 as lead SNP (β = −0.018, p = 2.87x10^-7^). This SNP is in the coding sequence of *DMD*. Dysregulation in *DMD* can cause Duchenne Muscular Dystrophy. Patients with this disease also show elevated cholesterol levels [59]. No reported eQTL or associated GWAS were identified. Thus, we could assign plausible candidate genes to all of our suggestive loci.

### Replication of reported associations and look-up of other cardiovascular trait associations

We looked-up four SNP sets that are reported for associations with ABI, PAD, CAD, and carotid plaque presence. Results are shown in S4 Table in S1 File. A SNP association was considered successfully replicated, respectively co-associated with other cardiovascular disease traits, if it showed the expected effect direction (same for ABI, opposite for PAD, CAD, and Carotid plaque burden) and a one-sided p-value below 0.05 (liberal) or FDR < 0.05 (stringent) (Table 3).

**Table 3:** Validation of reported associations and look-up of other CVD associations.

Reported trait association	Number of reported literature SNPs	Number of independent SNPs analysed	N SNPs replication level p < 0.05^$^	N SNPs replication level FDR < 0.05
ABI	14	6	1 (16.67%)	1 (rs10757269 -CDKN2B, PubMed ID 22199011)
PAD	149	24	4 (16.67%)*	0
CAD	2,557	342	28 (8.19%)*	2 (rs186696265, intergenic, PubMed ID 29212778; rs896655, intergenic, PubMed ID 29212778)
Plaque	52	13	2 (15.38%)	1 (rs9644862, CDKN2B-AS1, CDKN2B, PubMed ID 28282560)

For replication we compared our results with the unique published SNPs that had the lowest p-value across reported studies. We only consider data with identifiable effect direction. Details on replicated SNPs is provided in S4 table in S1 File, overlap of respective cytobands is shown in S6 Fig ^$^ one sided p-value <0.05. *Significantly more than 5%, what would be expected by chance (binomial testing with p_expected_≤0.05).

*ABI:* Of the 14 unique SNPs reported for association with ABI, six were reported with effect allele and beta estimate and corresponded to unique SNPs or proxies in our replication analysis. Of these, all had power above 80% (see S4 Table in S1 File for details) and we were able to validate rs10757269 (9 p 21.3), which also showed an FDR below 0.05. The 9p21.3 locus is a well-known CAD locus, linked to regulation of cell proliferation [60].

*PAD:* Of the 149 unique SNPs reported for association with PAD, 24 were reported with effect allele and beta estimate allowing our validation analysis. Of these, 16 had power above 80% and we were able to validate four of them at nominal level. Rs118039278 (6q25.3, *LPA* locus), rs1537372 (again 9p21.3, *CDKN2B-AS1* locus), rs4722172 (7p15.3, *IL6*-locus) and rs6025 (1q24.2, Factor V Leiden locus) are all PAD-associated SNPs previously reported in a large population study (the Million Veteran Program with replication in the UK Biobank, [3]). However, none of these associations achieved an FDR below 0.05.

*CAD:* Our analysis of 342 independent CAD-associated SNPs revealed nominal significant ABI associations for 28 variants, exceeding what would be expected by chance (p_Enrich _= 0.0079). Two of these SNPs achieved significant association at FDR ≤ 0.05: the intergenic variants rs186696265 (6q26) and rs896655 (again 9p21.3). Both variants were previously identified in a large-scale cardiovascular study, the UK Biobank with replication in CARDIoGRAMplusC4D [30]. The 6q26 locus (*LPA*) was also found in our ABI meta-GWAS.

*Carotid plaque:* In our examination of carotid plaque-associated variants (using an expanded phenotype definition detailed in the supplement), we found nominal ABI associations for two out of 13 independent SNPs. These included rs9644862 at the established risk locus 9p21 and rs1528152 at 7p21, with the latter having previously shown only suggestive evidence in the reporting study (p_original study_ = 3 × 10^−7^) [61]. The 9p21 association remained significant at FDR ≤ 0.05. Notably, 9p21 emerged as the only locus showing association evidence across all four analysed phenotypes: ABI, PAD, CAD, and carotid plaque underlining its high relevance for atherosclerosis development (S6 Fig).

### Transcriptome-wide association meta-analysis (TWAMA)

**Fig 2:**
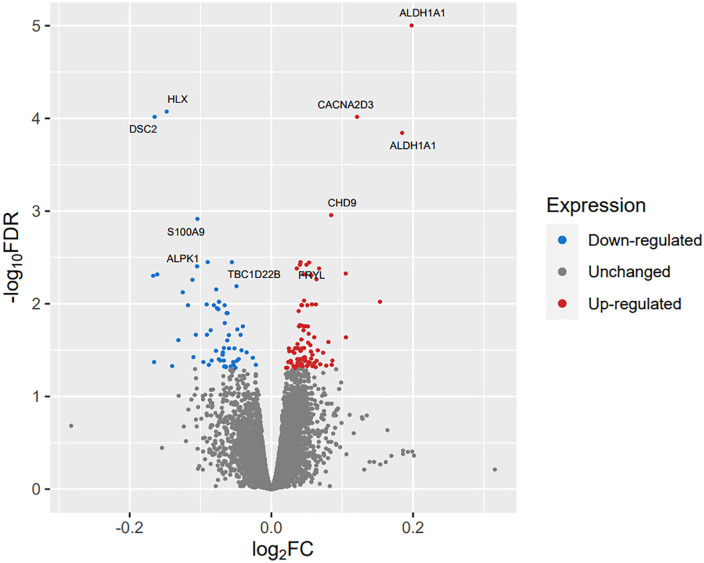
Volcano plot showing the association between measured gene-expression in blood and ABI. Fold changes and adjusted p-values were computed in the fixed-effect meta-analysis of the LIFE-Adult- and LIFE-Heart cohorts. We assigned gene names to the top ten associations.

Next, we investigated genome-wide gene expression association in a TWAMA including measured gene expression data from the LIFE-Adult- and LIFE-Heart cohort. This identified 152 transcripts, representing 145 genes, showing significant (FDR < 0.05) positive (93) or negative (59) association with ABI. Statistics of significant associations are shown in S5 Table in S1 File and in Fig 2. For example, two probes of ALDH1A1 were among the top associations showing the strongest positive correlation with ABI.

**Fig 3:**
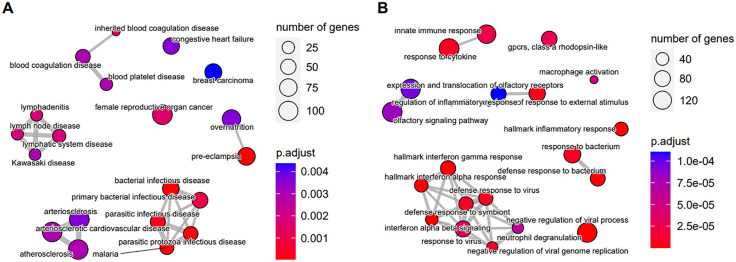
Enrichment plot of the top 20 significantly associating gene sets showing negative association with the ABI (i.e., increased expression in patients with PAD) from genome-wide results of TWAMA. A) Gene sets from the Disease Ontology, B) gene sets collated from several databases of pathways. The placement of the gene sets and thickness of the connecting lines reflects the degree of similarity of the gene sets, i.e., number of common genes.

**Fig 4:**
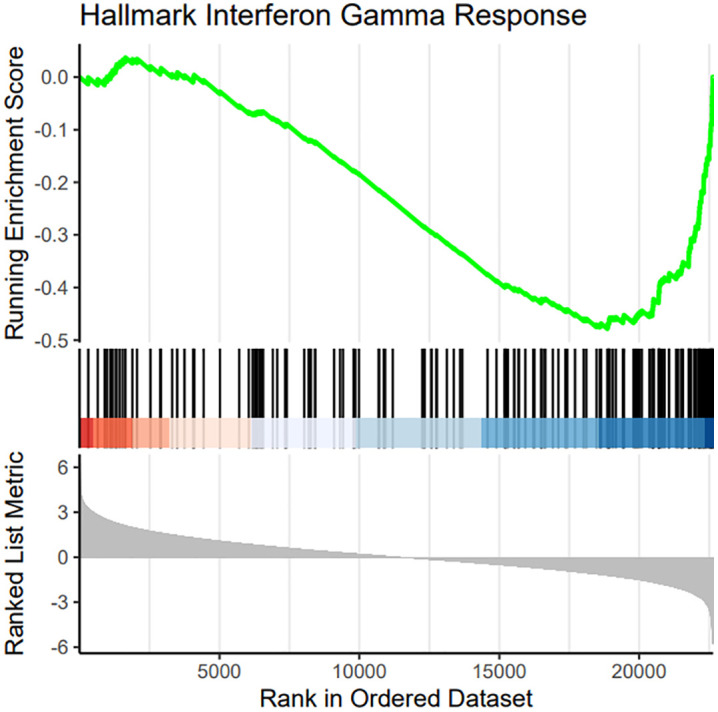
Gene Set Enrichment plot of the gene set representing the interferon gamma response pathway. This gene set is most strongly enriched at the bottom of the list of genes ranked by their association with the ABI, i.e., shows a strong negative association with the ABI (Benjamini-Hochberg adjusted p = 1.4x10^-8,^ Normalized Enrichment Score NES = −2.4).

Fast Gene Set Enrichment Analysis (FGSEA) applied to the results of the TWAMA identified 3 respectively 46 Disease Ontology terms (DOSE Ontology) as significantly (with adjusted p-value < 0.05) associated with an increase respectively decrease in ABI. Among the latter were terms as “atherosclerosis”, “arteriosclerotic cardiovascular disease”, “congestive heart failure” and “blood coagulation disease” (S6 Table in S1 File, Fig 3A). In addition, we performed FGSEA of a comprehensive collection of gene sets representing various signalling pathways (Fig 3B). This analysis yielded 371 and 175 gene sets showing positive and negative correlation with the ABI, respectively (S7 Table in S1 File). Gene sets most strongly associated with a decrease of the ABI were related to interferon-signalling, e.g., hallmark interferon gamma response (Fig 4).

We performed an enrichment analysis testing whether genes within 1 Mb of the GWAS top hits for ankle-brachial index (ABI) are enriched among genes showing significant differential expression (FDR < 0.05) in the TWAMA. Among 16 genes located within 1 Mb of the four GWAS loci, one gene (MAP3K4) also showed significant differential expression in the TWAMA analysis. While this represents a 2.2-fold enrichment compared to the expected rate (6.2% observed vs 2.8% expected), the overlap was not statistically significant (Fisher’s exact test p = 0.37, OR = 2.28, 95% CI: 0.05–14.88).

### Genetically regulated gene-expression association analysis

Based on genetically controlled gene-expression following the S-PrediXcan framework, we identified 832 gene-expressions of artery tissues (tibial, aorta or coronary) and 586 gene-expressions of whole blood to be associated with ABI with nominal significance p-value < 0.05 in S-PrediXcan. However, none of these associations achieved an FDR value < 0.05. FGSEA analysis of association results from arterial tissues and whole blood did not reveal significant enrichment of gene sets from either Disease Ontology or signalling pathway collections (S8 Table in S1 File).

Nevertheless, when we stratified the results of S-PrediXcan, our analysis offered additional insight beyond the established cross-tissue correlations of eQTLs. This was achieved by demonstrating the specific concordance between the genetically determined baseline expression levels (as identified by S-PrediXcan) and the observed phenotypic variation in our study. This finding highlights how the heritable component of gene expression is directly associated with the phenotype of interest. Identified overlapping genes between artery tissues and whole blood are shown in S9 Table in S1 File.

**Fig 5:**
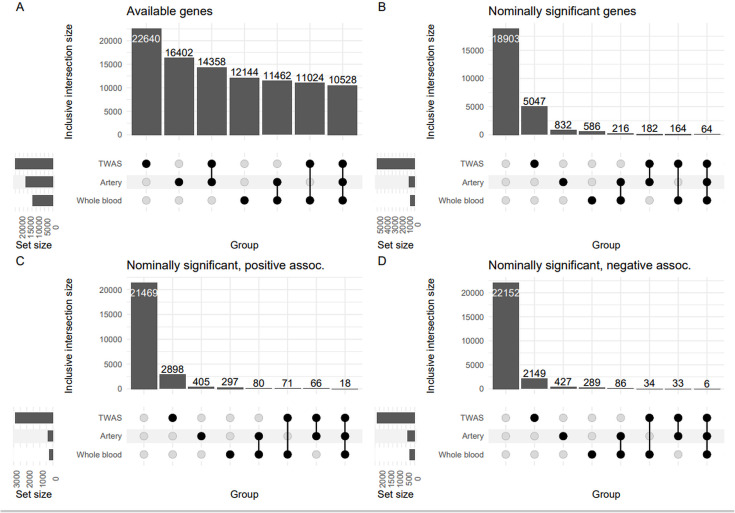
Upset plots showing numbers of genes that meet specific conditions in our three gene expression analyse. 1) tissue-based gene expression analysis of the arterial tissue, 2) tissue-based gene expression analysis of whole blood, and 3) transcriptome-wide association meta-analysis (TWAMA). A) numbers of available genes. B) numbers of nominally significant associating genes. C) and D) numbers of nominally significant associating genes showing a positive or negative association with the ABI, respectively. The shown intersections are inclusive, i.e., genes that belong to the sets defining an intersection may overlap with other sets if available. Details of overlapping genes is shown in S10 Table in S1 File.

We then combined these results with our findings from TWAMA. Fig 5 shows the overlap of associated genes identified in the three gene expression analyses: 1) tissue-based gene expression analysis of the arterial tissue (S-PrediXcan), 2) tissue-based gene expression analysis of the whole blood (S-PrediXcan), and 3) in the transcriptome-wide association meta-analysis of directly measured gene expression. We identified 6 consistently negative and 18 consistently positive correlated genes with ABI at nominal significance level in all three analyses (Table 4, all genes are shown in S10 Table in S1 File).

**Table 4:** Overlapping of nominally associated genes in blood TWAMA, blood S-PrediXcan and artery tissue S-PrediXcan analysis with consistent effect size.

Gene symbol	Z-score from arterial tissue S-PrediXcan	P-value from arterial tissue S-PrediXcan	Z-score from whole blood S-PrediXcan	P-value from whole blood S-PrediXcan	Z-score from measured expression TWAMA	P-value from measured expression TWAMA
NDUFB6	−2.43	3.2x10^-02^	−2.22	2.7x10^-02^	−3.04	2.3x10^-03^
PPP1R15A	−2.73	4.0x10^-02^	−2.73	6.4x10^-03^	−2.44	1.5x10^-02^
TGM2	−2.25	2.4x10^-02^	−2.25	2.4x10^-02^	−2.38	1.7x10^-02^
PCNX1	−3.27	2.4x10^-03^	−2.07	3.9x10^-02^	−2.21	2.7x10^-02^
GCLM	−2.46	4.9x10^-02^	−2.38	1.7x10^-02^	−2.13	3.3x10^-02^
CCM2	−2.75	2.7x10^-02^	−2.40	1.6x10^-02^	−2.05	4.0x10^-02^
GOLT1B	2.99	1.1x10^-02^	2.25	2.4x10^-02^	1.96	5.0x10^-02^
KDELC2	1.97	4.9x10^-02^	1.97	4.9x10^-02^	1.97	4.9x10^-02^
GALNT11	2.26	2.9x10^-02^	2.26	2.4x10^-02^	2.06	3.9x10^-02^
COPS6	2.37	5.0x10^-02^	2.37	1.8x10^-02^	2.09	3.7x10^-02^
RETSAT	3.30	1.0x10^-02^	2.89	3.8x10^-03^	2.12	3.4x10^-02^
TXNDC16	3.20	1.4x10^-03^	3.22	1.3x10^-03^	2.13	3.3x10^-02^
PRRC1	2.83	4.1x10^-03^	2.34	1.9x10^-02^	2.18	3.0x10^-02^
CYTH1	2.47	3.9x10^-02^	2.47	1.4x10^-02^	2.20	2.8x10^-02^
ALDH7A1	1.93	4.8x10^-02^	2.37	1.8x10^-02^	2.33	2.0x10^-02^
LRRC23	2.17	3.1x10^-02^	2.01	4.5x10^-02^	2.37	1.8x10^-02^
PKIG	2.51	1.3x10^-02^	2.51	1.2x10^-02^	2.60	9.2x10^-03^
CEP192	2.32	2.4x10^-02^	2.22	2.6x10^-02^	2.61	8.9x10^-03^
GGACT	2.44	2.7x10^-02^	2.19	2.8x10^-02^	2.67	7.5x10^-03^
NIFK	2.36	1.8x10^-02^	2.36	1.8x10^-02^	3.03	2.4x10^-03^
SNX16	2.50	4.2x10^-02^	2.30	2.1x10^-02^	3.15	1.6x10^-03^
TRMT61B	2.64	8.8x10^-03^	2.64	8.3x10^-03^	3.37	7.5x10^-04^
RABL2B	2.67	9.5x10^-03^	2.54	1.1x10^-02^	4.05	5.1x10^-05^
RAD51C	3.14	7.1x10^-03^	3.14	1.7x10^-03^	4.17	3.1x10^-05^

A total of 24 genes showed nominally significant associations in TWAMA as well as in artery tissue and whole blood. Overlap of all analysed genes is shown in S10 Table in S1 File.

The genes negatively correlated with ABI in all three analyses prominently feature in cellular stress response and vascular integrity pathways. Key examples include *GCLM*, central to glutathione synthesis and cellular antioxidant defence [62], and *PPP1R15A* (*GADD34*), which regulates the integrated stress response [63]. CCM2 is crucial for endothelial stability and vascular integrity [64]. *TGM2*’s involvement in vascular stiffness and inflammation [65] further underscores the connection between these genes and vascular health.

In contrast, the genes positively correlated with ABI primarily cluster around pathways essential for protein homeostasis, cellular trafficking, and metabolic regulation. This group includes genes involved in protein processing and transport (*GOLT1B*, *KDELC2*), protein glycosylation (*GALNT11*), and cellular quality control (*COPS6* as subunit of the *COP9* signalosome complex). Additionally, *RETSAT* plays a role in retinol metabolism, which is significant for vascular health due to vitamin A’s role in cell differentiation [66]. Several genes in this group also contribute to cellular integrity, such as *ALDH7A1* in cellular detoxification and *RAD51C* in DNA repair and genomic stability.

Pathway analysis of the 24 overlapping genes using g:Profiler revealed significant enrichment in cellular modified amino acid biosynthetic processes (e.g., involving GCLM and ALDH7A1, Fold Enrichment: 34.8, p-value: 0.001) and cellular modified amino acid metabolic processes (Fold Enrichment: 12.4, p-value: 0.002). Additionally, rRNA processing (Fold Enrichment: 6.9, p-value: 0.033) and rRNA metabolic processes (Fold Enrichment: 5.9, p-value: 0.044), involving genes like NIFK and TRMT61B, were significantly enriched (see S11 Table in S1 File for details).

Finally, we analysed gene-expression levels in human heart left ventricle tissue of the 24 overlapping genes (S7 Fig). This supports several ABI candidate genes, e.g., *CCM2*, *TGM2*, and *GCLM* given their roles in vascular integrity, inflammation, and oxidative stress responses and their expression in vascular endothelial and immune cells.

## Discussion

In our study, we performed a comprehensive integrative analysis of genetic and transcriptomic factors influencing ABI. The results contribute to the understanding of the genetic architecture of ABI and identify a multitude of potential new genes affecting vascular health.

In our genetic meta-analysis of two cohorts no significant inflation of the test statistic was observed, indicating reliable results and efficient data pre-processing. While no genome-wide significant associations were found, we identified four suggestive loci to which we can assign biologically plausible candidate genes. In our TWAMA one probe of *DMD* showed nominal significance (p = 0.046). The association with CAD relevant loci (*LPA* and *DLG2*) needs further evaluation to better understand the underlying mechanisms of action. For *TMTC2* and *DMD* the cause and effect is well understood due to their involvement in cholesterol metabolism. TMTC2 localizes to the endoplasmic reticulum where it regulates calcium homeostasis and protein folding. Its association with HDL cholesterol suggests a role in lipid metabolism, possibly through ER stress pathways. DMD maintains sarcolemmal integrity in muscle and vascular smooth muscle cells. While best known for Duchenne muscular dystrophy, DMD variants also influence cholesterol metabolism and vascular stiffness. The X-linked inheritance of DMD may contribute to sex differences in PAD risk. Together, these genes implicate cellular stress responses, lipid metabolism, and vascular integrity in ABI determination [67,68].

We furthermore validated previously reported SNPs for their associations with ABI, PAD, CAD, and carotid plaque burden. While we were not able to identify SNPs with genome-wide significance for ABI in our data, we replicated one ABI and four PAD SNPs and identified co-association of 28 CAD and two plaque SNPs at nominal level. These are more associations at this level than expected by chance, supporting genetic commonalities between these different vascular endpoints.

Measured blood gene-expression association analysis identified 145 differentially expressed genes at FDR 5% level. The fact that we identified more direct associations with measured gene-expression than with analysis of genetic regulated tissue specific expression (S-PrediXcan) suggests that context specific gene-expression is more relevant than genetically regulated gene-expression. The limited overlap between TWAMA and S- PrediXcan may also reflects fundamental differences in what these approaches capture. S-PrediXcan identifies genetically determined baseline expression levels, representing the heritable component of gene expression [69]. In contrast, TWAMA captures the full transcriptional response including environmental factors, inflammation, disease state, and medication effects. The stronger associations in TWAMA, particularly for inflammatory pathways, suggest that acquired changes in gene expression play a larger role in ABI variation than constitutive genetic differences. This is consistent with PAD pathophysiology, where inflammatory responses to vascular injury may be more important than baseline expression levels [35]. Additionally, S-PrediXcan models are trained in healthy tissues, potentially missing disease-specific regulatory relationships. The 24 genes showing consistent associations across both approaches likely represent core pathways where both genetic predisposition and environmental response converge. Pathway analysis of all TWAMA genes resulted in plausible pathways and points towards interferon-signalling as a potential driving factor for PAD molecular pathology. The strong association between interferon gamma response pathways and ABI identified in our analysis is in line with existing research [70]. In a recent study, hallmark interferon gamma response was one of the most significantly enriched pathways in macrophages from symptomatic human atherosclerotic plaques [71].

We combined TWAMA results to integrate information from measured gene expression data with tissue-specific, genetic driven expression changes. Overlapping genes at nominal association significance were also plausible: Genes with expression levels correlating with lower ABI values were associated with increased activation of stress response pathways and compromised vascular integrity [62–65], while genes with expression levels correlating with higher ABI values were related to enhanced cellular homeostasis, efficient protein quality control mechanisms, and metabolic regulation [66] (Table 4 and S10 Table in S1 File). This underscores the value of the overlapping genes as candidate genes for pathomechanistical follow up-studies, to confirm that in vascular health, compromised circulation can involve cellular stress and altered protein homeostasis, whereas robust cellular function can support healthy vascular tissue.

We acknowledge several limitations of our study: 1) We investigated a limited sample size as our meta GWAS included a total of 9,662 people and lacked an independent replication cohort. However, we provided full summary statistics of each analysed cohort enabling future meta-analyses to confirm suggestive loci identified by us. 2) We only considered European subjects, hence, generalisation of our findings to other ethnicities needs to be explored in future studies. 3) We could not validate identified top-genes in functional studies, however, we provide reported functional evidence where available. 4) Comprehensive, retrospective electronic health record (EHR) data for defining cardiovascular conditions such as CAD and PAD was not uniformly available across both cohorts. Specifically, for the population-based LIFE-Adult study, EHR data was only available prospectively. While for the LIFE-Heart clinical cohort, definite CAD diagnosis was based on coronary angiography and PAD diagnosis relied on ABI cutoffs and clinical diagnostics, our approach for certain definitions, such as PAD based on ABI cutoffs, was thus utilized. Future work leveraging more extensive and consistently available EHR data across diverse cohorts could enable a more directly clinically-based assessment of these conditions. 5) A further limitation of our study stems from the use of different blood pressure measurement methodologies across the two cohorts. The LIFE-Adult cohort employed oscillometry for blood pressure measurements, while the LIFE-Heart cohort utilized Doppler ultrasound. These methods, while both measuring systolic blood pressure ratios, can yield slightly different absolute values. This methodological disparity is known to translate into phenotypic differences in Ankle-Brachial Index (ABI) values, as previously demonstrated [72]. To address this potential source of phenotypic heterogeneity and ensure the robustness of our findings, we opted for a meta-analysis approach, combining statistical results from both cohorts rather than directly pooling the raw data. This strategy allowed us to account for the inherent differences in measurement techniques between the cohorts while still leveraging the combined power of the studies.

In conclusion, while no genome-wide significant ABI-associated SNPs emerged, our integrative genetic and transcriptomic analyses revealed suggestive signals and identified genes whose expression correlates strongly with ABI. The enrichment of nominally significant overlapping genes in both arterial tissues (including the aorta) and whole blood, and their directional consistency with ABI, highlights systemic regulatory mechanisms underlying peripheral arterial health. Our findings point toward pathways involved in cellular stress response, vascular integrity, metabolic regulation, and interferon-signalling as potential contributors to ABI variation. These results provide a foundation for future investigations aiming to understand the molecular determinants of vascular function and peripheral arterial disease risk.

## Supporting information

S1 Fig Principal component analysis of first two principal components for LIFE-Adult and LIFE-Heart. (TIF)

S2 Fig Integrated principal component analysis of first two principal components for LIFE-Adult and LIFE-Heart. Displaying the cases and controls together within the same PC space for each cohort. The PC space was defined using reference samples from the HapMap project, representing three major continental populations: African (AFR), Asian (ASN), and European (EUR).
(TIF)

S3 Fig Sensitivity analyses comparing the primary GWAS meta-analysis results with two alternative approaches. Each point represents the effect estimate of one of the four top SNPs. The error bars show the SE from the association meta-analysis. A) Comparison between the main analysis and an analysis excluding one sample of related pairs (N = 9488; 174 related individuals removed). Relatedness was defined as exceeding a relatedness threshold > 0.2, corresponding to second-degree relatives. B) Comparison between the main analysis and an analysis including all individuals but adjusting for the first 10 genetic principal components. Pearson correlation coefficients indicate high concordance between approaches: r = 0.9980 in panel A and r = 0.9996 in panel B.
(TIF)

S4 Fig QQ Plot showing the observed quantiles vs the expected Chi-squared statistic. The factor lambda was 0.999, i.e., not indicating any evidence for inflation of test statistics. Red circles indicate SNPs with minor allele frequency < 0.05.
(TIF)

S5 Fig Regional ABI association plots for our four top SNPs. The top SNPs are coloured blue, the other SNPs according to their LD with the respective lead SNP). The top SNPs are always supported by additional variants.
(TIF)

S6 Fig Upset plots showing numbers of cytobands that meet specific conditions A) All variants, B) Nominally significant variants, C) Associations significant at FDR 5%. The shown intersections are inclusive, i.e., genes that belong to the sets defining an intersection may overlap with other sets if available.
(TIF)

S7 Fig Pathway and gene-set enrichment analysis specifically on the 24 genes consistently associated across both our TWAMA and S-PrediXcan approaches. This analysis shows significant enrichment in several biologically relevant pathways.
(TIF)

S1 File S Tables. Supplementary Tables 1–12.
(XLSX)

S2 File Supplementary Methods. Technical supplementary data and supplementary references.
(DOCX)
