# Supplementary figures and images for: Genome- and transcriptome-wide association meta-analysis reveals new insights into genes affecting coronary and peripheral artery disease
Source: PLoS One. 2025 Nov 18;20(11):e0335513. doi: 10.1371/journal.pone.0335513 (PMC12626291; doi:10.1371/journal.pone.0335513)

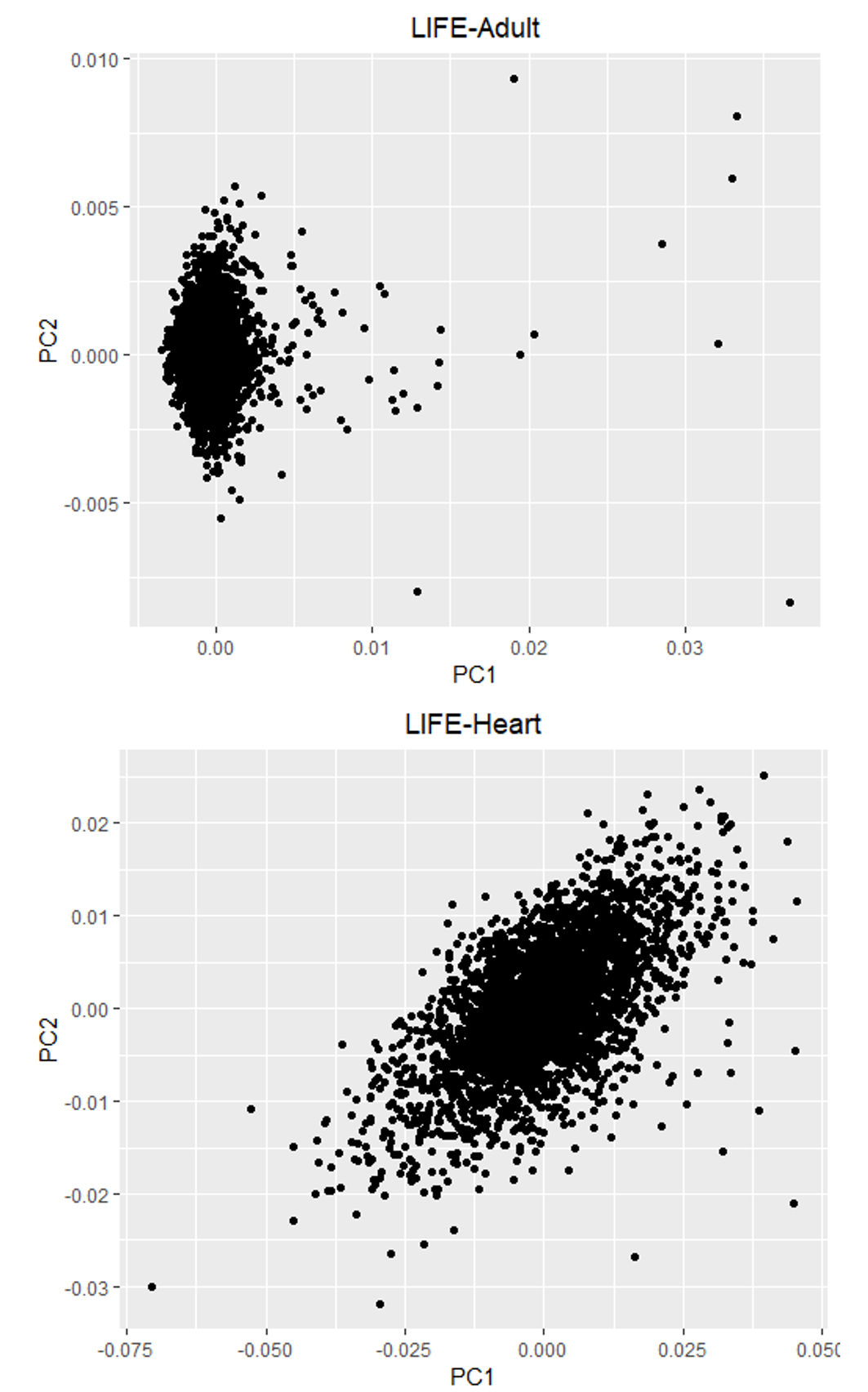

Supplement: S1 Fig — (TIF) [file pone.0335513.s001.tif]

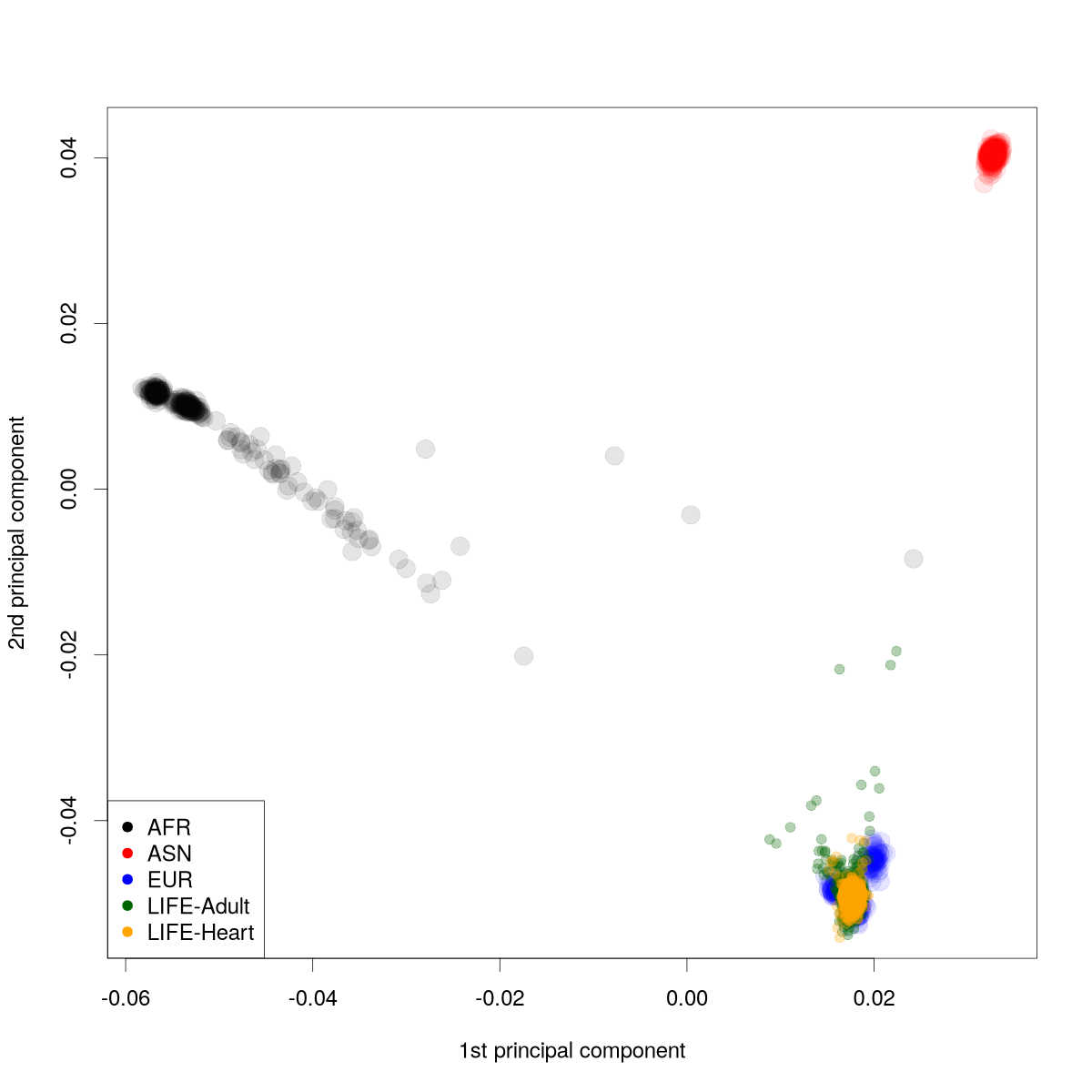

Supplement: S2 Fig — Displaying the cases and controls together within the same PC space for each cohort. The PC space was defined using reference samples from the HapMap project, representing three major continental populations: African (AFR), Asian (ASN), and European (EUR). (TIF) [file pone.0335513.s002.tif]

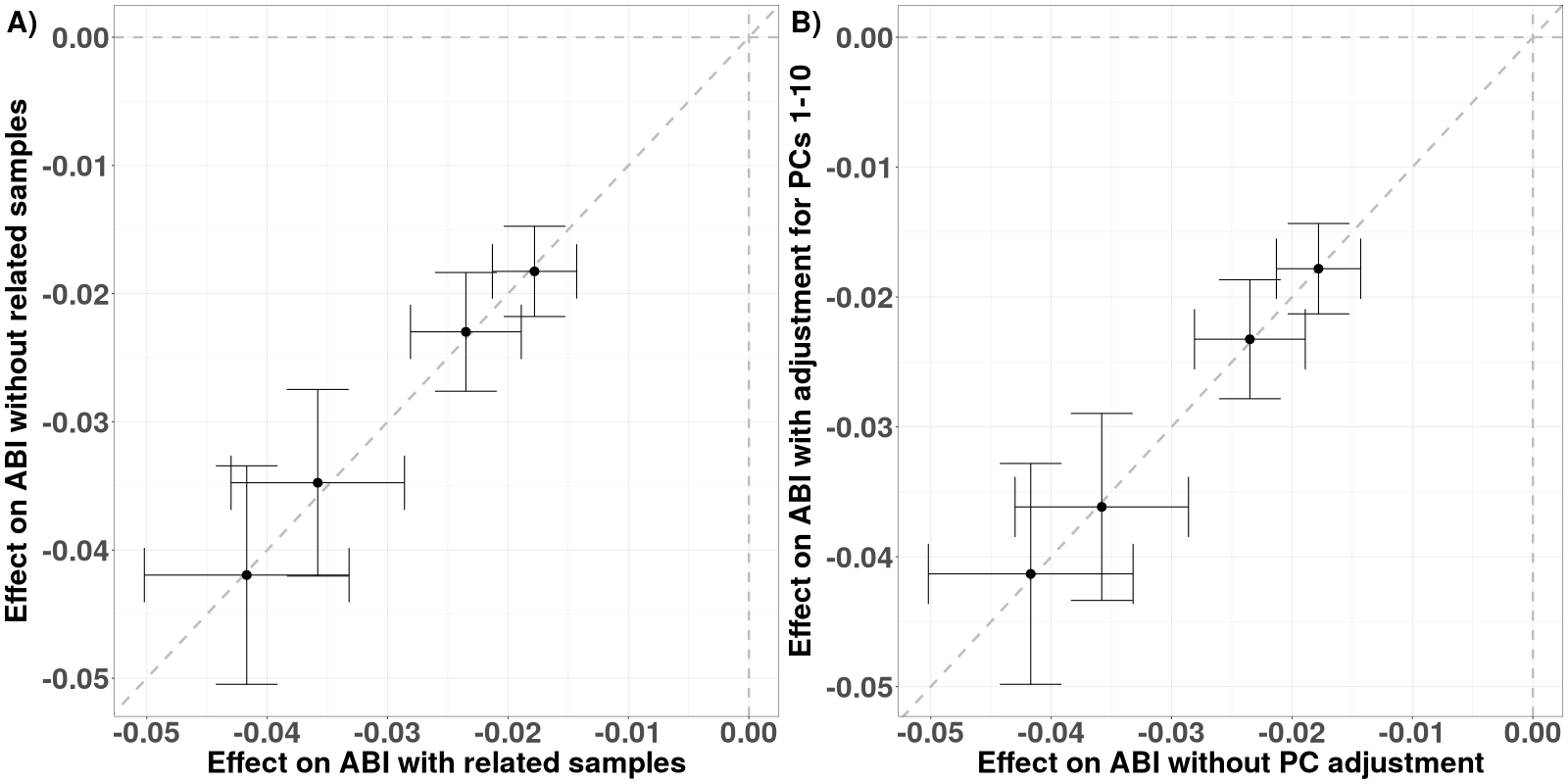

Supplement: S3 Fig — Each point represents the effect estimate of one of the four top SNPs. The error bars show the SE from the association meta-analysis. A) Comparison between the main analysis and an analysis excluding one sample of related pairs (N = 9488; 174 related individuals removed). Relatedness was defined as exceeding a relatedness threshold > 0.2, corresponding to second-degree relatives. B) Comparison between the main analysis and an analysis including all individuals but adjusting for the first 10 genetic principal components. Pearson correlation coefficients indicate high concordance between approaches: r = 0.9980 in panel A and r = 0.9996 in panel B. (TIF) [file pone.0335513.s003.tif]

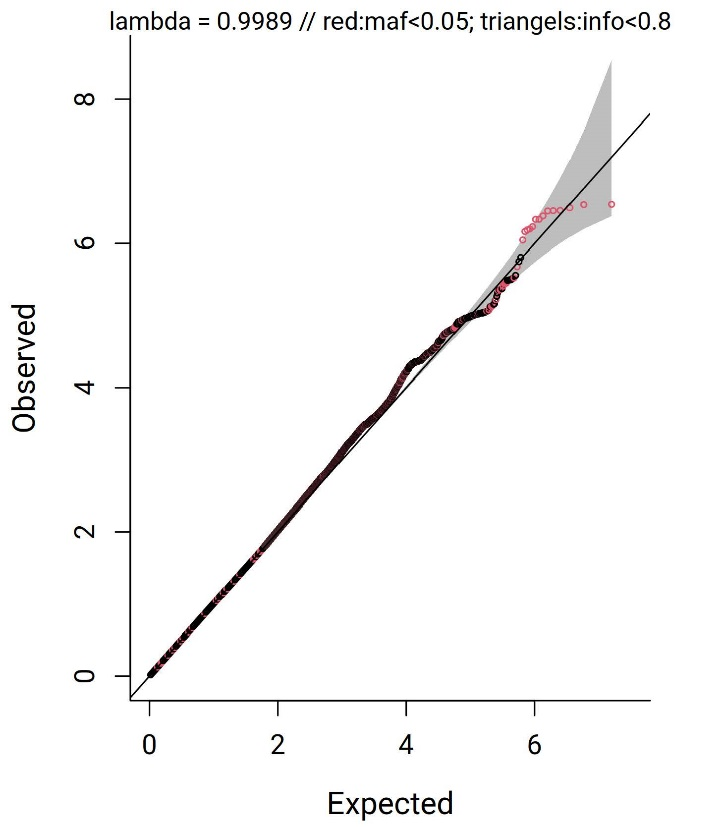

Supplement: S4 Fig — The factor lambda was 0.999, i.e., not indicating any evidence for inflation of test statistics. Red circles indicate SNPs with minor allele frequency < 0.05. (TIF) [file pone.0335513.s004.tif]

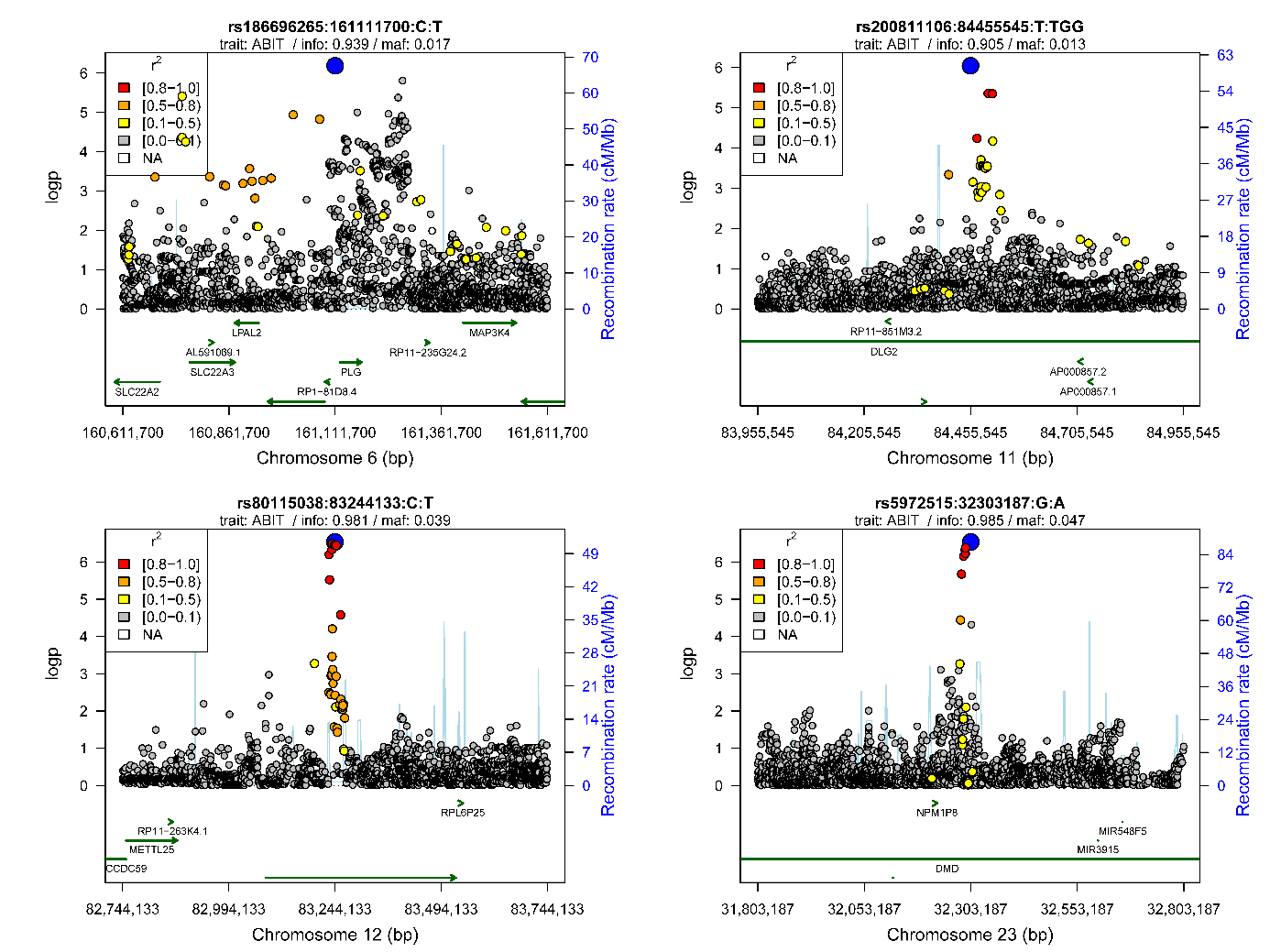

Supplement: S5 Fig — The top SNPs are coloured blue, the other SNPs according to their LD with the respective lead SNP). The top SNPs are always supported by additional variants. (TIF) [file pone.0335513.s005.tif]

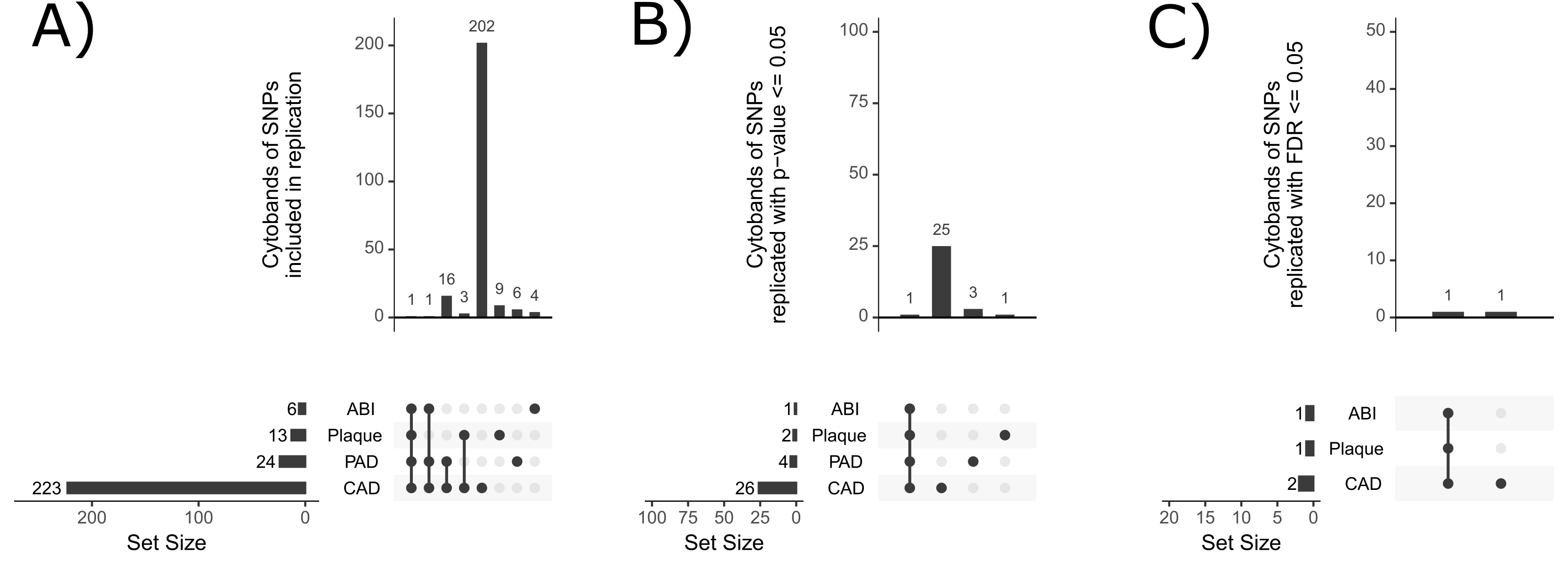

Supplement: S6 Fig — The shown intersections are inclusive, i.e., genes that belong to the sets defining an intersection may overlap with other sets if available. (TIF) [file pone.0335513.s006.tif]

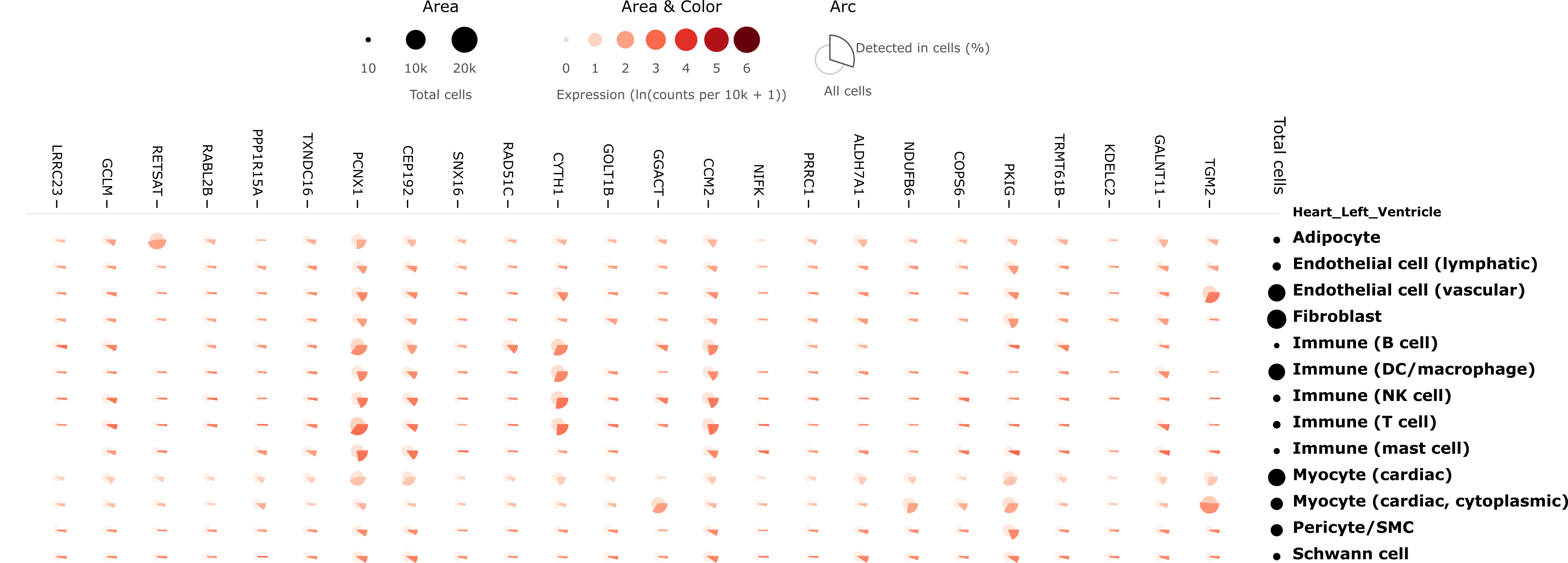

Supplement: S7 Fig — This analysis shows significant enrichment in several biologically relevant pathways. (TIF) [file pone.0335513.s007.tif]
