# Supplementary material for: Genome- and transcriptome-wide association meta-analysis reveals new insights into genes affecting coronary and peripheral artery disease
Source: PLoS One. 2025 Nov 18;20(11):e0335513. doi: 10.1371/journal.pone.0335513 (PMC12626291; doi:10.1371/journal.pone.0335513)
Supplement: S2 File — Technical supplementary data and supplementary references. (DOCX) [file pone.0335513.s009.docx]

**Supplementary Methods**

The GWAS catalogue was downloaded in 2022, Dec, 15, 16, 16, 21, for CAD, PAD, Plaque, and ABI-related phenotypes, respectively. Reported association with p‑value > 10^-6^ were not considered.

Phenotypes included in carotid plaque burden:

Sum of carotid plaque area

Carotid plaque maximum area

Mean area of carotid plaque

Maximum stenosis

Sum of stenosis

References used to identify candidate genes are as follows:

ABI [1–4],

CAD [5–56],

PAD [2,3,19,46,57–62]

References

1. O'Donnell CJ, Cupples LA, D'Agostino RB, Fox CS, Hoffmann U, Hwang S-J, Ingellson E, Liu C, Murabito JM, Polak JF, Wolf PA, Demissie S: **Genome-wide association study for subclinical atherosclerosis in major arterial territories in the NHLBI's Framingham Heart Study**. *BMC medical genetics* 2007, **8 Suppl 1**:S4.

2. Sofer T, Emery L, Jain D, Ellis AM, Laurie CC, Allison MA, Lee J, Kurniansyah N, Kerr KF, González HM, Tarraf W, Criqui MH, Lange LA, Palmas WR, Franceschini N, Wassel CL: **Variants Associated with the Ankle Brachial Index Differ by Hispanic/Latino Ethnic Group: a genome-wide association study in the Hispanic Community Health Study/Study of Latinos**. *Scientific reports* 2019, **9**:11410.

3. Klarin D, Lynch J, Aragam K, Chaffin M, Assimes TL, Huang J, Lee KM, Shao Q, Huffman JE, Natarajan P, Arya S, Small A, Sun YV, Vujkovic M, Freiberg MS, Wang L, Chen J, Saleheen D, Lee JS, Miller DR, Reaven P, Alba PR, Patterson OV, DuVall SL, Boden WE, Beckman JA, Gaziano JM, Concato J, Rader DJ, Cho K *et al.*: **Genome-wide association study of peripheral artery disease in the Million Veteran Program**. *Nature medicine* 2019, **25**:1274-1279.

4. Murabito JM, White CC, Kavousi M, Sun YV, Feitosa MF, Nambi V, Lamina C, Schillert A, Coassin S, Bis JC, Broer L, Crawford DC, Franceschini N, Frikke-Schmidt R, Haun M, Holewijn S, Huffman JE, Hwang S-J, Kiechl S, Kollerits B, Montasser ME, Nolte IM, Rudock ME, Senft A, Teumer A, van der Harst P, Vitart V, Waite LL, Wood AR, Wassel CL *et al.*: **Association between chromosome 9p21 variants and the ankle-brachial index identified by a meta-analysis of 21 genome-wide association studies**. *Circulation. Cardiovascular genetics* 2012, **5**:100-112.

5. Antikainen AAV, Sandholm N, Trégouët D-A, Charmet R, McKnight AJ, Ahluwalia TS, Syreeni A, Valo E, Forsblom C, Gordin D, Harjutsalo V, Hadjadj S, Maxwell AP, Rossing P, Groop P-H: **Genome-wide association study on coronary artery disease in type 1 diabetes suggests beta-defensin 127 as a risk locus**. *Cardiovascular research* 2021, **117**:600-612.

6. Charmet R, Duffy S, Keshavarzi S, Gyorgy B, Marre M, Rossing P, McKnight AJ, Maxwell AP, Ahluwalia TVS, Paterson AD, Trégouët D-A, Hadjadj S: **Novel risk genes identified in a genome-wide association study for coronary artery disease in patients with type 1 diabetes**. *Cardiovascular diabetology* 2018, **17**:61.

7. **A genome-wide association study in Europeans and South Asians identifies five new loci for coronary artery disease**. *Nature genetics* 2011, **43**:339-344.

8. Davies RW, Wells GA, Stewart AFR, Erdmann J, Shah SH, Ferguson JF, Hall AS, Anand SS, Burnett MS, Epstein SE, Dandona S, Chen L, Nahrstaedt J, Loley C, König IR, Kraus WE, Granger CB, Engert JC, Hengstenberg C, Wichmann H-E, Schreiber S, Tang WHW, Ellis SG, Rader DJ, Hazen SL, Reilly MP, Samani NJ, Schunkert H, Roberts R, McPherson R: **A genome-wide association study for coronary artery disease identifies a novel susceptibility locus in the major histocompatibility complex**. *Circulation. Cardiovascular genetics* 2012, **5**:217-225.

9. Dehghan A, Bis JC, White CC, Smith AV, Morrison AC, Cupples LA, Trompet S, Chasman DI, Lumley T, Völker U, Buckley BM, Ding J, Jensen MK, Folsom AR, Kritchevsky SB, Girman CJ, Ford I, Dörr M, Salomaa V, Uitterlinden AG, Eiriksdottir G, Vasan RS, Franceschini N, Carty CL, Virtamo J, Demissie S, Amouyel P, Arveiler D, Heckbert SR, Ferrières J *et al.*: **Genome-Wide Association Study for Incident Myocardial Infarction and Coronary Heart Disease in Prospective Cohort Studies: The CHARGE Consortium**. *PloS one* 2016, **11**:e0144997.

10. Dichgans M, Malik R, König IR, Rosand J, Clarke R, Gretarsdottir S, Thorleifsson G, Mitchell BD, Assimes TL, Levi C, O'Donnell CJ, Fornage M, Thorsteinsdottir U, Psaty BM, Hengstenberg C, Seshadri S, Erdmann J, Bis JC, Peters A, Boncoraglio GB, März W, Meschia JF, Kathiresan S, Ikram MA, McPherson R, Stefansson K, Sudlow C, Reilly MP, Thompson JR, Sharma P *et al.*: **Shared genetic susceptibility to ischemic stroke and coronary artery disease: a genome-wide analysis of common variants**. *Stroke* 2014, **45**:24-36.

11. Domarkienė I, Pranculis A, Germanas S, Jakaitienė A, Vitkus D, Dženkevičiūtė V, Kučinskienė Z, Kučinskas V: **RTN4 and FBXL17 Genes are Associated with Coronary Heart Disease in Genome-Wide Association Analysis of Lithuanian Families**. *Balkan journal of medical genetics: BJMG* 2013, **16**:17-22.

12. Erdmann J, Grosshennig A, Braund PS, König IR, Hengstenberg C, Hall AS, Linsel-Nitschke P, Kathiresan S, Wright B, Trégouët D-A, Cambien F, Bruse P, Aherrahrou Z, Wagner AK, Stark K, Schwartz SM, Salomaa V, Elosua R, Melander O, Voight BF, O'Donnell CJ, Peltonen L, Siscovick DS, Altshuler D, Merlini PA, Peyvandi F, Bernardinelli L, Ardissino D, Schillert A, Blankenberg S *et al.*: **New susceptibility locus for coronary artery disease on chromosome 3q22.3**. *Nature genetics* 2009, **41**:280-282.

13. Erdmann J, Willenborg C, Nahrstaedt J, Preuss M, König IR, Baumert J, Linsel-Nitschke P, Gieger C, Tennstedt S, Belcredi P, Aherrahrou Z, Klopp N, Loley C, Stark K, Hengstenberg C, Bruse P, Freyer J, Wagner AK, Medack A, Lieb W, Grosshennig A, Sager HB, Reinhardt A, Schäfer A, Schreiber S, El Mokhtari NE, Raaz-Schrauder D, Illig T, Garlichs CD, Ekici AB *et al.*: **Genome-wide association study identifies a new locus for coronary artery disease on chromosome 10p11.23**. *European heart journal* 2011, **32**:158-168.

14. Fall T, Gustafsson S, Orho-Melander M, Ingelsson E: **Genome-wide association study of coronary artery disease among individuals with diabetes: the UK Biobank**. *Diabetologia* 2018, **61**:2174-2179.

15. Han Y, Dorajoo R, Chang X, Wang L, Khor C-C, Sim X, Cheng C-Y, Shi Y, Tham YC, Zhao W, Chee ML, Sabanayagam C, Chee ML, Tan N, Wong TY, Tai E-S, Liu J, Goh DYT, Yuan J-M, Koh W-P, van Dam RM, Low AF, Chan MY-Y, Friedlander Y, Heng C-K: **Genome-wide association study identifies a missense variant at APOA5 for coronary artery disease in Multi-Ethnic Cohorts from Southeast Asia**. *Scientific reports* 2017, **7**:17921.

16. He L, Kernogitski Y, Kulminskaya I, Loika Y, Arbeev KG, Loiko E, Bagley O, Duan M, Yashkin A, Ukraintseva SV, Kovtun M, Yashin AI, Kulminski AM: **Pleiotropic Meta-Analyses of Longitudinal Studies Discover Novel Genetic Variants Associated with Age-Related Diseases**. *Frontiers in genetics* 2016, **7**:179.

17. Huang LO, Rauch A, Mazzaferro E, Preuss M, Carobbio S, Bayrak CS, Chami N, Wang Z, Schick UM, Yang N, Itan Y, Vidal-Puig A, Hoed M den, Mandrup S, Kilpeläinen TO, Loos RJF: **Genome-wide discovery of genetic loci that uncouple excess adiposity from its comorbidities**. *Nature metabolism* 2021, **3**:228-243.

18. Huertas-Vazquez A, Nelson CP, Guo X, Reinier K, Uy-Evanado A, Teodorescu C, Ayala J, Jerger K, Chugh H, Braund PS, Deloukas P, Hall AS, Balmforth AJ, Jones M, Taylor KD, Pulit SL, Newton-Cheh C, Gunson K, Jui J, Rotter JI, Albert CM, Samani NJ, Chugh SS: **Novel loci associated with increased risk of sudden cardiac death in the context of coronary artery disease**. *PloS one* 2013, **8**:e59905.

19. Ishigaki K, Akiyama M, Kanai M, Takahashi A, Kawakami E, Sugishita H, Sakaue S, Matoba N, Low S-K, Okada Y, Terao C, Amariuta T, Gazal S, Kochi Y, Horikoshi M, Suzuki K, Ito K, Koyama S, Ozaki K, Niida S, Sakata Y, Sakata Y, Kohno T, Shiraishi K, Momozawa Y, Hirata M, Matsuda K, Ikeda M, Iwata N, Ikegawa S *et al.*: **Large-scale genome-wide association study in a Japanese population identifies novel susceptibility loci across different diseases**. *Nature genetics* 2020, **52**:669-679.

20. Klarin D, Zhu QM, Emdin CA, Chaffin M, Horner S, McMillan BJ, Leed A, Weale ME, Spencer CCA, Aguet F, Segrè AV, Ardlie KG, Khera AV, Kaushik VK, Natarajan P, Kathiresan S: **Genetic analysis in UK Biobank links insulin resistance and transendothelial migration pathways to coronary artery disease**. *Nature genetics* 2017, **49**:1392-1397.

21. Koyama S, Ito K, Terao C, Akiyama M, Horikoshi M, Momozawa Y, Matsunaga H, Ieki H, Ozaki K, Onouchi Y, Takahashi A, Nomura S, Morita H, Akazawa H, Kim C, Seo J-S, Higasa K, Iwasaki M, Yamaji T, Sawada N, Tsugane S, Koyama T, Ikezaki H, Takashima N, Tanaka K, Arisawa K, Kuriki K, Naito M, Wakai K, Suna S *et al.*: **Population-specific and trans-ancestry genome-wide analyses identify distinct and shared genetic risk loci for coronary artery disease**. *Nature genetics* 2020, **52**:1169-1177.

22. Kulminski AM, Huang J, Loika Y, Arbeev KG, Bagley O, Yashkin A, Duan M, Culminskaya I: **Strong impact of natural-selection-free heterogeneity in genetics of age-related phenotypes**. *Aging* 2018, **10**:492-514.

23. Lee J-Y, Lee B-S, Shin D-J, Woo Park K, Shin Y-A, Joong Kim K, Heo L, Young Lee J, Kyoung Kim Y, Jin Kim Y, Bum Hong C, Lee S-H, Yoon D, Jung Ku H, Oh I-Y, Kim B-J, Lee J, Park S-J, Kim J, Kawk H-K, Lee J-E, Park H-K, Lee J-E, Nam H-Y, Park H-Y, Shin C, Yokota M, Asano H, Nakatochi M, Matsubara T *et al.*: **A genome-wide association study of a coronary artery disease risk variant**. *Journal of human genetics* 2013, **58**:120-126.

24. Lettre G, Palmer CD, Young T, Ejebe KG, Allayee H, Benjamin EJ, Bennett F, Bowden DW, Chakravarti A, Dreisbach A, Farlow DN, Folsom AR, Fornage M, Forrester T, Fox E, Haiman CA, Hartiala J, Harris TB, Hazen SL, Heckbert SR, Henderson BE, Hirschhorn JN, Keating BJ, Kritchevsky SB, Larkin E, Li M, Rudock ME, McKenzie CA, MEIGS JB, Meng YA *et al.*: **Genome-wide association study of coronary heart disease and its risk factors in 8,090 African Americans: the NHLBI CARe Project**. *PLoS genetics* 2011, **7**:e1001300.

25. Li Y, Wang DW, Chen Y, Chen C, Guo J, Zhang S, Sun Z, Ding H, Yao Y, Zhou L, Xu K, Song C, Yang F, Zhao B, Yan H, Wang W-J, Wu C, Lu X, Yang X, Dong J, Zheng G, Tian S, Cui Y, Jin L, Liu G, Cui H, Wang S, Jiang F, Wang C, Erdmann J *et al.*: **Genome-Wide Association and Functional Studies Identify SCML4 and THSD7A as Novel Susceptibility Genes for Coronary Artery Disease**. *Arteriosclerosis, thrombosis, and vascular biology* 2018, **38**:964-975.

26. Liu Y, Ma H, Zhu Q, Zhang B, Yan H, Li H, Meng J, Lai W, Li L, Yu D, Zhong S: **A genome-wide association study on lipoprotein (a) levels and coronary artery disease severity in a Chinese population**. *Journal of lipid research* 2019, **60**:1440-1448.

27. Lu X, Wang L, Chen S, He L, Yang X, Shi Y, Cheng J, Zhang L, Gu CC, Huang J, Wu T, Ma Y, Li J, Cao J, Chen J, Ge D, Fan Z, Li Y, Zhao L, Li H, Zhou X, Chen L, Liu D, Chen J, Duan X, Hao Y, Wang L, Lu F, Liu Z, Yao C *et al.*: **Genome-wide association study in Han Chinese identifies four new susceptibility loci for coronary artery disease**. *Nature genetics* 2012, **44**:890-894.

28. Matsunaga H, Ito K, Akiyama M, Takahashi A, Koyama S, Nomura S, Ieki H, Ozaki K, Onouchi Y, Sakaue S, Suna S, Ogishima S, Yamamoto M, Hozawa A, Satoh M, Sasaki M, Yamaji T, Sawada N, Iwasaki M, Tsugane S, Tanaka K, Arisawa K, Ikezaki H, Takashima N, Naito M, Wakai K, Tanaka H, Sakata Y, Morita H, Sakata Y *et al.*: **Transethnic Meta-Analysis of Genome-Wide Association Studies Identifies Three New Loci and Characterizes Population-Specific Differences for Coronary Artery Disease**. *Circulation. Genomic and precision medicine* 2020, **13**:e002670.

29. Nelson CP, Goel A, Butterworth AS, Kanoni S, Webb TR, Marouli E, Zeng L, Ntalla I, Lai FY, Hopewell JC, Giannakopoulou O, Jiang T, Hamby SE, Di Angelantonio E, Assimes TL, Bottinger EP, Chambers JC, Clarke R, Palmer CNA, Cubbon RM, Ellinor P, Ermel R, Evangelou E, Franks PW, Grace C, Gu D, Hingorani AD, Howson JMM, Ingelsson E, Kastrati A *et al.*: **Association analyses based on false discovery rate implicate new loci for coronary artery disease**. *Nature genetics* 2017, **49**:1385-1391.

30. Nikpay M, Goel A, Won H-H, Hall LM, Willenborg C, Kanoni S, Saleheen D, Kyriakou T, Nelson CP, Hopewell JC, Webb TR, Zeng L, Dehghan A, Alver M, Armasu SM, Auro K, Bjonnes A, Chasman DI, Chen S, Ford I, Franceschini N, Gieger C, Grace C, Gustafsson S, Huang J, Hwang S-J, Kim YK, Kleber ME, Lau KW, Lu X *et al.*: **A comprehensive 1,000 Genomes-based genome-wide association meta-analysis of coronary artery disease**. *Nature genetics* 2015, **47**:1121-1130.

31. Reilly MP, Li M, He J, Ferguson JF, Stylianou IM, Mehta NN, Burnett MS, Devaney JM, Knouff CW, Thompson JR, Horne BD, Stewart AFR, Assimes TL, Wild PS, Allayee H, Nitschke PL, Patel RS, Martinelli N, Girelli D, Quyyumi AA, Anderson JL, Erdmann J, Hall AS, Schunkert H, Quertermous T, Blankenberg S, Hazen SL, Roberts R, Kathiresan S, Samani NJ *et al.*: **Identification of ADAMTS7 as a novel locus for coronary atherosclerosis and association of ABO with myocardial infarction in the presence of coronary atherosclerosis: two genome-wide association studies**. *Lancet (London, England)* 2011, **377**:383-392.

32. Samani NJ, Erdmann J, Hall AS, Hengstenberg C, Mangino M, Mayer B, Dixon RJ, Meitinger T, Braund P, Wichmann H-E, Barrett JH, König IR, Stevens SE, Szymczak S, Tregouet D-A, Iles MM, Pahlke F, Pollard H, Lieb W, Cambien F, Fischer M, Ouwehand W, Blankenberg S, Balmforth AJ, Baessler A, Ball SG, Strom TM, Braenne I, Gieger C, Deloukas P *et al.*: **Genomewide association analysis of coronary artery disease**. *The New England journal of medicine* 2007, **357**:443-453.

33. Saw J, Yang M-L, Trinder M, Tcheandjieu C, Xu C, Starovoytov A, Birt I, Mathis MR, Hunker KL, Schmidt EM, Jackson L, Fendrikova-Mahlay N, Zawistowski M, Brummett CM, Zoellner S, Katz A, Coleman DM, Swan K, O'Donnell CJ, Zhou X, Li JZ, Gornik HL, Assimes TL, Stanley JC, Brunham LR, Ganesh SK: **Chromosome 1q21.2 and additional loci influence risk of spontaneous coronary artery dissection and myocardial infarction**. *Nature communications* 2020, **11**:4432.

34. Schmitz B, Kleber ME, Lenders M, Delgado GE, Engelbertz C, Huang J, Pavenstädt H, Breithardt G, Brand S-M, März W, Brand E: **Genome-wide association study suggests impact of chromosome 10 rs139401390 on kidney function in patients with coronary artery disease**. *Scientific reports* 2019, **9**:2750.

35. Schunkert H, König IR, Kathiresan S, Reilly MP, Assimes TL, Holm H, Preuss M, Stewart AFR, Barbalic M, Gieger C, Absher D, Aherrahrou Z, Allayee H, Altshuler D, Anand SS, Andersen K, Anderson JL, Ardissino D, Ball SG, Balmforth AJ, Barnes TA, Becker DM, Becker LC, Berger K, Bis JC, Boekholdt SM, Boerwinkle E, Braund PS, Brown MJ, Burnett MS *et al.*: **Large-scale association analysis identifies 13 new susceptibility loci for coronary artery disease**. *Nature genetics* 2011, **43**:333-338.

36. Shiffman D, Trompet S, Louie JZ, Rowland CM, Catanese JJ, Iakoubova OA, Kirchgessner TG, Westendorp RGJ, Craen AJM de, Slagboom PE, Buckley BM, Stott DJ, Sattar N, Devlin JJ, Packard CJ, Ford I, Sacks FM, Jukema JW: **Genome-wide study of gene variants associated with differential cardiovascular event reduction by pravastatin therapy**. *PloS one* 2012, **7**:e38240.

37. Siewert KM, Voight BF: **Bivariate Genome-Wide Association Scan Identifies 6 Novel Loci Associated With Lipid Levels and Coronary Artery Disease**. *Circulation. Genomic and precision medicine* 2018, **11**:e002239.

38. Slavin TP, Feng T, Schnell A, Zhu X, Elston RC: **Two-marker association tests yield new disease associations for coronary artery disease and hypertension**. *Human genetics* 2011, **130**:725-733.

39. Song Y, Choi J-E, Kwon Y-J, Chang H-J, Kim JO, Park D-H, Park J-M, Kim S-J, Lee JW, Hong K-W: **Identification of susceptibility loci for cardiovascular disease in adults with hypertension, diabetes, and dyslipidemia**. *Journal of translational medicine* 2021, **19**:85.

40. Takeuchi F, Yokota M, Yamamoto K, Nakashima E, Katsuya T, Asano H, Isono M, Nabika T, Sugiyama T, Fujioka A, Awata N, Ohnaka K, Nakatochi M, Kitajima H, Rakugi H, Nakamura J, Ohkubo T, Imai Y, Shimamoto K, Yamori Y, Yamaguchi S, Kobayashi S, Takayanagi R, Ogihara T, Kato N: **Genome-wide association study of coronary artery disease in the Japanese**. *European journal of human genetics: EJHG* 2012, **20**:333-340.

41. Tcheandjieu C, Zhu X, Hilliard AT, Clarke SL, Napolioni V, Ma S, Lee KM, Fang H, Chen F, Lu Y, Tsao NL, Raghavan S, Koyama S, Gorman BR, Vujkovic M, Klarin D, Levin MG, Sinnott-Armstrong N, Wojcik GL, Plomondon ME, Maddox TM, Waldo SW, Bick AG, Pyarajan S, Huang J, Song R, Ho Y-L, Buyske S, Kooperberg C, Haessler J *et al.*: **Large-scale genome-wide association study of coronary artery disease in genetically diverse populations**. *Nature medicine* 2022, **28**:1679-1692.

42. Temprano-Sagrera G, Sitlani CM, Bone WP, Martin-Bornez M, Voight BF, Morrison AC, Damrauer SM, Vries PS de, Smith NL, Sabater-Lleal M: **Multi-phenotype analyses of hemostatic traits with cardiovascular events reveal novel genetic associations**. *Journal of thrombosis and haemostasis: JTH* 2022, **20**:1331-1349.

43. Trégouët D-A, König IR, Erdmann J, Munteanu A, Braund PS, Hall AS, Grosshennig A, Linsel-Nitschke P, Perret C, DeSuremain M, Meitinger T, Wright BJ, Preuss M, Balmforth AJ, Ball SG, Meisinger C, Germain C, Evans A, Arveiler D, Luc G, Ruidavets J-B, Morrison C, van der Harst P, Schreiber S, Neureuther K, Schäfer A, Bugert P, El Mokhtari NE, Schrezenmeir J, Stark K *et al.*: **Genome-wide haplotype association study identifies the SLC22A3-LPAL2-LPA gene cluster as a risk locus for coronary artery disease**. *Nature genetics* 2009, **41**:283-285.

44. van der Harst P, Verweij N: **Identification of 64 Novel Genetic Loci Provides an Expanded View on the Genetic Architecture of Coronary Artery Disease**. *Circulation research* 2018, **122**:433-443.

45. van Zuydam NR, Ladenvall C, Voight BF, Strawbridge RJ, Fernandez-Tajes J, Rayner NW, Robertson NR, Mahajan A, Vlachopoulou E, Goel A, Kleber ME, Nelson CP, Kwee LC, Esko T, Mihailov E, Mägi R, Milani L, Fischer K, Kanoni S, Kumar J, Song C, Hartiala JA, Pedersen NL, Perola M, Gieger C, Peters A, Qu L, Willems SM, Doney ASF, Morris AD *et al.*: **Genetic Predisposition to Coronary Artery Disease in Type 2 Diabetes Mellitus**. *Circulation. Genomic and precision medicine* 2020, **13**:e002769.

46. Vujkovic M, Keaton JM, Lynch JA, Miller DR, Zhou J, Tcheandjieu C, Huffman JE, Assimes TL, Lorenz K, Zhu X, Hilliard AT, Judy RL, Huang J, Lee KM, Klarin D, Pyarajan S, Danesh J, Melander O, Rasheed A, Mallick NH, Hameed S, Qureshi IH, Afzal MN, Malik U, Jalal A, Abbas S, Sheng X, Gao L, Kaestner KH, Susztak K *et al.*: **Discovery of 318 new risk loci for type 2 diabetes and related vascular outcomes among 1.4 million participants in a multi-ancestry meta-analysis**. *Nature genetics* 2020, **52**:680-691.

47. Wakil SM, Ram R, Muiya NP, Mehta M, Andres E, Mazhar N, Baz B, Hagos S, Alshahid M, Meyer BF, Morahan G, Dzimiri N: **A genome-wide association study reveals susceptibility loci for myocardial infarction/coronary artery disease in Saudi Arabs**. *Atherosclerosis* 2016, **245**:62-70.

48. Wang Z, Zhu Q, Liu Y, Chen S, Zhang Y, Ma Q, Chen X, Liu C, Lei H, Chen H, Wang J, Zheng S, Li Z, Xiong L, Lai W, Zhong S: **Genome-wide association study of metabolites in patients with coronary artery disease identified novel metabolite quantitative trait loci**. *Clinical and translational medicine* 2021, **11**:e290.

49. **Genome-wide association study of 14,000 cases of seven common diseases and 3,000 shared controls**. *Nature* 2007, **447**:661-678.

50. Westermair AL, Munz M, Schaich A, Nitsche S, Willenborg B, Muñoz Venegas LM, Willenborg C, Schunkert H, Schweiger U, Erdmann J: **Association of Genetic Variation at AQP4 Locus with Vascular Depression**. *Biomolecules* 2018, **8**.

51. Wild PS, Zeller T, Schillert A, Szymczak S, Sinning CR, Deiseroth A, Schnabel RB, Lubos E, Keller T, Eleftheriadis MS, Bickel C, Rupprecht HJ, Wilde S, Rossmann H, Diemert P, Cupples LA, Perret C, Erdmann J, Stark K, Kleber ME, Epstein SE, Voight BF, Kuulasmaa K, Li M, Schäfer AS, Klopp N, Braund PS, Sager HB, Demissie S, Proust C *et al.*: **A genome-wide association study identifies LIPA as a susceptibility gene for coronary artery disease**. *Circulation. Cardiovascular genetics* 2011, **4**:403-412.

52. Yamada Y, Yasukochi Y, Kato K, Oguri M, Horibe H, Fujimaki T, Takeuchi I, Sakuma J: **Identification of 26 novel loci that confer susceptibility to early-onset coronary artery disease in a Japanese population**. *Biomedical reports* 2018, **9**:383-404.

53. Yeo A, Li L, Warren L, Aponte J, Fraser D, King K, Johansson K, Barnes A, MacPhee C, Davies R, Chissoe S, Tarka E, O'Donoghue ML, White HD, Wallentin L, Waterworth D: **Pharmacogenetic meta-analysis of baseline risk factors, pharmacodynamic, efficacy and tolerability endpoints from two large global cardiovascular outcomes trials for darapladib**. *PloS one* 2017, **12**:e0182115.

54. Zhou W, Nielsen JB, Fritsche LG, Dey R, Gabrielsen ME, Wolford BN, LeFaive J, VandeHaar P, Gagliano SA, Gifford A, Bastarache LA, Wei W-Q, Denny JC, Lin M, Hveem K, Kang HM, Abecasis GR, Willer CJ, Lee S: **Efficiently controlling for case-control imbalance and sample relatedness in large-scale genetic association studies**. *Nature genetics* 2018, **50**:1335-1341.

55. Zhu Z, Wang X, Li X, Lin Y, Shen S, Liu C-L, Hobbs BD, Hasegawa K, Liang L, Boezen HM, Camargo CA, Cho MH, Christiani DC: **Genetic overlap of chronic obstructive pulmonary disease and cardiovascular disease-related traits: a large-scale genome-wide cross-trait analysis**. *Respiratory research* 2019, **20**:64.

56. Zhuang Z, Yao M, Wong JYY, Liu Z, Huang T: **Shared genetic etiology and causality between body fat percentage and cardiovascular diseases: a large-scale genome-wide cross-trait analysis**. *BMC medicine* 2021, **19**:100.

57. Matsukura M, Ozaki K, Takahashi A, Onouchi Y, Morizono T, Komai H, Shigematsu H, Kudo T, Inoue Y, Kimura H, Hosaka A, Shigematsu K, Miyata T, Watanabe T, Tsunoda T, Kubo M, Tanaka T: **Genome-Wide Association Study of Peripheral Arterial Disease in a Japanese Population**. *PloS one* 2015, **10**:e0139262.

58. Koriyama H, Nakagami H, Katsuya T, Sugimoto K, Yamashita H, Takami Y, Maeda S, Kubo M, Takahashi A, Nakamura Y, Ogihara T, Rakugi H, Kaneda Y, Morishita R: **Identification of evidence suggestive of an association with peripheral arterial disease at the OSBPL10 locus by genome-wide investigation in the Japanese population**. *Journal of atherosclerosis and thrombosis* 2010, **17**:1054-1062.

59. Kullo IJ, Shameer K, Jouni H, Lesnick TG, Pathak J, Chute CG, Andrade M de: **The ATXN2-SH2B3 locus is associated with peripheral arterial disease: an electronic medical record-based genome-wide association study**. *Frontiers in genetics* 2014, **5**:166.

60. Ward-Caviness CK, Neas LM, Blach C, Haynes CS, LaRocque-Abramson K, Grass E, Dowdy E, Devlin RB, Diaz-Sanchez D, Cascio WE, Lynn Miranda M, Gregory SG, Shah SH, Kraus WE, Hauser ER: **Genetic Variants in the Bone Morphogenic Protein Gene Family Modify the Association between Residential Exposure to Traffic and Peripheral Arterial Disease**. *PloS one* 2016, **11**:e0152670.

61. van Zuydam NR, Stiby A, Abdalla M, Austin E, Dahlström EH, McLachlan S, Vlachopoulou E, Ahlqvist E, Di Liao C, Sandholm N, Forsblom C, Mahajan A, Robertson NR, Rayner NW, Lindholm E, Sinisalo J, Perola M, Kallio M, Weiss E, Price J, Paterson A, Klein B, Salomaa V, Palmer CNA, Groop P-H, Groop L, McCarthy MI, Andrade M de, Morris AP, Hopewell JC *et al.*: **Genome-Wide Association Study of Peripheral Artery Disease**. *Circulation. Genomic and precision medicine* 2021, **14**:e002862.

62. Sakaue S, Kanai M, Tanigawa Y, Karjalainen J, Kurki M, Koshiba S, Narita A, Konuma T, Yamamoto K, Akiyama M, Ishigaki K, Suzuki A, Suzuki K, Obara W, Yamaji K, Takahashi K, Asai S, Takahashi Y, Suzuki T, Shinozaki N, Yamaguchi H, Minami S, Murayama S, Yoshimori K, Nagayama S, Obata D, Higashiyama M, Masumoto A, Koretsune Y, Ito K *et al.*: **A cross-population atlas of genetic associations for 220 human phenotypes**. *Nature genetics* 2021, **53**:1415-1424.
